# The pathogen’s playbook in cancer: from oncogenesis to progression

**DOI:** 10.3389/fonc.2025.1737423

**Published:** 2026-01-13

**Authors:** Hongzhou Cai, Shaozhe Yang, Ruizi Wang, Ruixin Li, Yihan Liu, Ruzhou Chen, Yun Hu, Ziwei Li, Jinzhou Zheng, Xuan Sun, Guoren Zhou

**Affiliations:** 1 Department of Urology, Jiangsu Cancer Hospital & The Affiliated Cancer Hospital of Nanjing Medical University & Jiangsu Institute of Cancer Research, Nanjing, Jiangsu, China; 2 Department of Radiology, The Fourth School of Clinical Medicine, Nanjing Medical University, Nanjing, Jiangsu, China; 3 Department of Breast, Jiangsu Cancer Hospital & The Affiliated Cancer Hospital of Nanjing Medical University & Jiangsu Institute of Cancer Research, Nanjing, Jiangsu, China; 4 Department of Oncology, Jiangsu Cancer Hospital & The Affiliated Cancer Hospital of Nanjing Medical University & Jiangsu Institute of Cancer Research, Nanjing, Jiangsu, China

**Keywords:** cancer, clinical translation, direct oncogenic mechanisms, DNA damage, immune evasion, microorganisms, tumor microenvironment, tumor progression regulation

## Abstract

Globally, around 15–20% of cancers are linked to microbial infections, involving viruses, bacteria, and fungi, each with distinct pathogenic features. Advanced multi-omics technologies have confirmed tumor-specific microbial communities within tumor tissues. This review categorizes microbes by their action mechanisms in cancer biology: one group (e.g., *Helicobacter pylori*, HPV, *Aspergillus* species) directly induces tumorigenesis via DNA damage, repairing pathway disruption, oncoprotein expression, and carcinogenic metabolite production; the other group (e.g., *Fusobacterium nucleatum*, HIV, *Candida albicans*) modulates tumor progression by regulating the tumor microenvironment, enhancing tumor cell chemoresistance, or suppressing anti-tumor immunity. Additionally, this review also explores clinical translation potential, highlights research challenges, and proposes future directions to support precision oncology advancement.

## 1 Introduction

**Figure 1:**
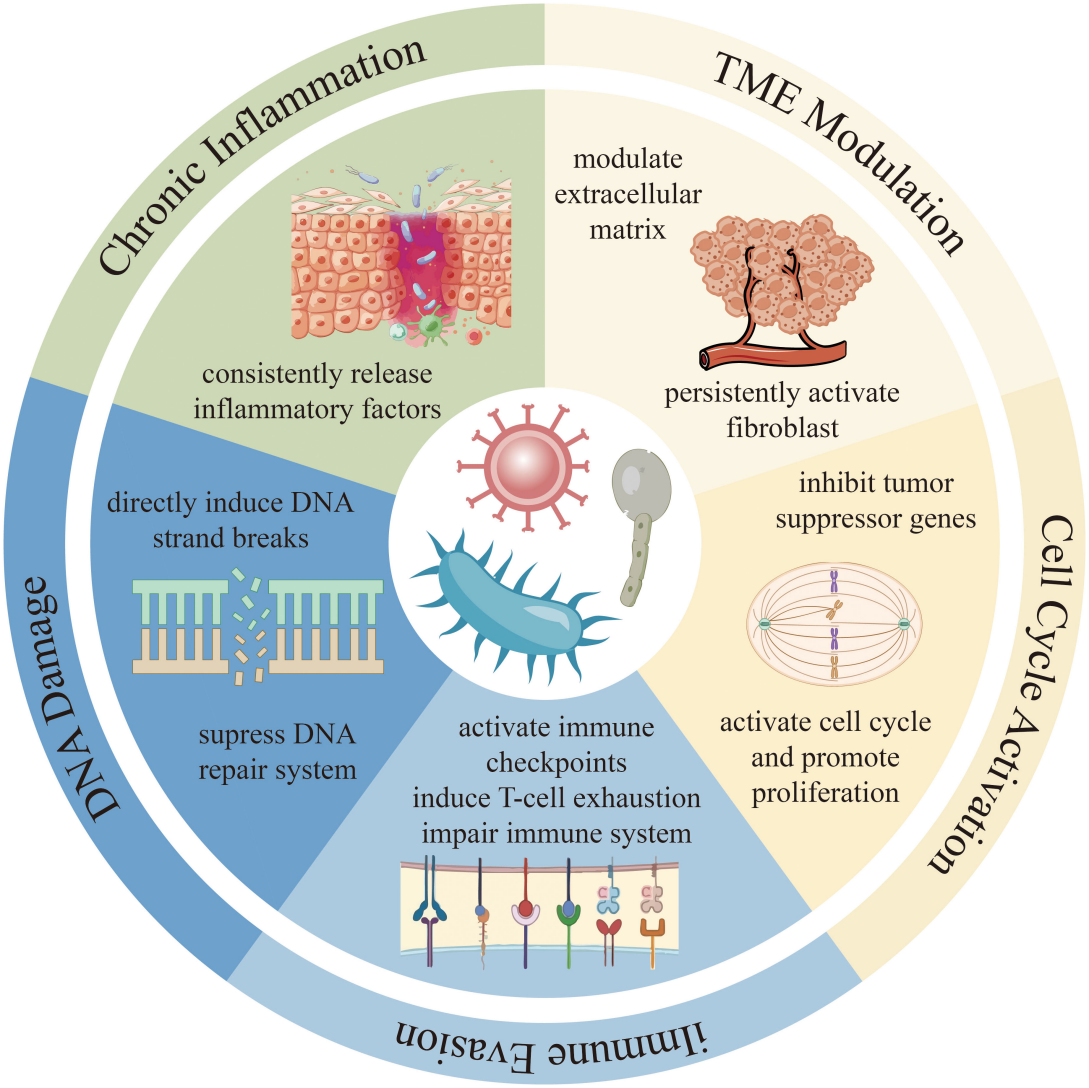
The common mechanisms which microorganisms use to directly or indirectly induce cancer.

In recent years, the intricate relationship between the microorganisms and cancer has emerged as a groundbreaking frontier in oncology. Traditionally regarded as passive bystanders or opportunistic invaders, microorganisms are now recognized as active participants in carcinogenesis. It is estimated that nearly 15–20% of global cancer cases are attributable to microbial infections, including viruses, bacteria, and fungi (1). Advanced multi-omics technologies have revealed that tumor tissues are not sterile, instead, they harbor diverse microbial communities that are often tumor-type specific. These findings challenge the classical one-pathogen-one-disease paradigm and suggest a model wherein polymicrobial ecosystems actively contribute to tumor initiation, progression, and response to therapy. As such, microorganisms are no longer viewed solely as cancer risk factors but as integral components of the tumor microenvironment (TME), influencing host immunity, inflammation, genomic stability and treatment resistance. **Figure 1** briefly summarizes some common mechanisms microorganisms employ to induce cancer.

Microorganisms are increasingly recognized as influential contributors to tumorigenesis, participating in cancer initiation and progression through diverse and complex mechanisms. Viruses, bacteria, and fungi each interact with the host in distinct ways, collectively shaping TME, altering immune responses, and affecting genomic stability. These interactions can promote malignant transformation, support tumor growth, and influence therapeutic outcomes. The involvement of microorganisms in cancer is not limited to a single pathway or effect but reflects a multifaceted biological interplay that varies across microorganisms and cancer contexts (2). Given the extensive and profound impact of microorganisms on cancer biology, this review aims to comprehensively elaborate on the mechanisms by which different microorganisms act in the occurrence and development of tumors, and to deeply explore how to develop innovative strategies for the prevention, diagnosis, and treatment of cancer based on these research insights.

## 2 Pathogens that directly cause tumors

Pathogens directly causing tumors, including bacteria, viruses, and fungi, exert carcinogenic effects through distinct mechanisms. Bacteria such as Helicobacter pylori and Salmonella Typhi induce DNA damage, subvert repair pathways, and trigger chronic inflammation to drive malignant transformation. Oncogenic viruses encode oncoproteins that disrupt cell cycle regulation, inhibit tumor suppressors, and promote genomic instability. Fungi like Aspergillus produce carcinogenic metabolites that induce critical gene mutations. These pathogens act as primary drivers of tumor initiation by directly altering host cellular processes.

### 2.1 Bacteria

Bacteria can directly drive human carcinogenesis via diverse mechanisms. The most prominent example is Helicobacter pylori, classified as a Group I carcinogen by the World Health Organization and a major risk factor for gastric cancer. More recently, other bacteria have been recognized as direct carcinogenic factors, such as genotoxin-producing pks^+^ Escherichia coli and enterotoxigenic Bacteroides fragilis (BFT), toxin-producing Clostridioides difficile (TcdB), and the gastric mucosa colonizer Streptococcus anginosus (3). The following section focuses on elucidating the specific molecular mechanisms of carcinogenesis employed by Helicobacter pylori and Salmonella Typhi.

#### 2.1.1 Helicobacter pylori

Helicobacter pylori(H.p) is a key risk factor for gastric cancer. Approximately 90% of gastric cancer cases can be attributed to H.p infection (4). In 2018, 812,000 cases of gastric cancer, including gastric non-Hodgkin lymphoma, were recorded, accounting for about 37% of all cancers driven by chronic infections. This makes H. pylori the most common carcinogenic pathogen (5). After H.p infection, chronic inflammation of the gastric mucosa will be triggered. It mediates the imbalance between cell damage and repair through chronic inflammation, thus leading to the occurrence and development of cancer. H. pylori acts on inflammatory cells, leading to a large accumulation of reactive oxygen species (ROS) and reactive nitrogen species (RNS) by increasing the expression of Nicotinamide adenine dinucleotide phosphate(NOX) and inducible nitric oxide synthase (6). Excessive production of ROS and RNS can lead to multiple DNA damage, such as point mutations, DNA adducts, and single- or double-strand breaks (DSB) (7). Under normal physiological conditions, the precisely regulated DNA Damage Response (DDR) network within cells promptly identifies and repairs such damage; if the damage cannot be repaired, this network initiates cell cycle arrest to ensure genomic stability or triggers the apoptosis program to eliminate damaged cells. However, the carcinogenic potential of H. pylori lies not only in its capacity to cause DNA damage but, more insidiously, in its ability to actively subvert these crucial DNA repair pathways. This subversion leads to inaccurate repair, genomic instability, and chromosomal aberrations, paving the way for malignant transformation. Central to this sabotage are the bacterium’s key virulence factors, the vacuolating cytotoxin A (VacA) and the cytotoxin-associated gene A (CagA) protein.

The VacA complex can integrate into the host cell membrane, functioning as an anionic selection channel. As a channel, VacA facilitates the release of bicarbonate and organic anions into the host cell cytoplasm, thereby aiding H. pylori colonization by providing metabolic substrates for bacterial growth (8). Exogenous VacA can target different organelles within host cells. For instance, the VacA complex can enter endosomes via endocytosis, and it has been suggested that VacA applied extracellularly can target mitochondria, and it induces the release of cytochrome C and apoptosis. Additionally, VacA exposure, particularly with NH4Cl, triggers ER stress that induces C/EBP homologous protein via eIF2-alpha kinase, causing mitochondrial impairment and apoptosis (9, 10).

The CagA protein is a primary driver of H. p-mediated pathogenesis. It is encoded within a 40-kb genomic region known as the cag pathogenicity island, which also contains the genes for the type IV secretion system molecular syringe used for its translocation into host cells. A primary mechanism by which CagA undermines genomic integrity is through the widespread downregulation of genes integral to multiple DNA repair pathways. This suppression is not random but appears to target key components across the DDR network. This table elucidates the impact of CagA on DNA repair pathways (**Table 1**) and clarifies the relevant molecular mechanisms.

**Table 1:** Regulatory effects of CagA on DNA repair pathways.

Repair pathway	Affected gene/protein	Change	Findings	References
Homologous Recombination (HR)	RAD54L, POLD1, POLD2, TOP3A	Downregulation	CagA downregulates the expression of these genes, thereby compromising high-fidelity repair of DNA double-strand breaks (DSBs).	(11)
	MRE11,RAD51	Downregulation		(11, 12)
Base Excision Repair (BER)	NTHL1,MUTYH, LIG1	Downregulation	Reduced protein levels impair the recognition and repair of DNA damage.	(11)
	APE1	Up-regulation	Consistently increased during infection, potentially reflecting dysregulation of the BER pathway rather than mere inhibition.	(11)
Mismatch Repair (MMR)	MLH1, MSH2, MSH6	Downregulation	Core component downregulation leads to microsatellite instability (MSI) and the accumulation of mutations	(11, 13)
	POLD1, POLD2, POLD3, LIG1	Downregulation	impacting DNA synthesis and ligation	
Nucleotide Excision Repair (NER)	DDB1, XPD	Downregulation	Downregulation of key genes involved in damage recognition and DNA unwinding leads to impairment of the NER pathway.	(11)
	POLD1, POLD2, POLD3, POLE, LIG1	Downregulation	Downregulation of DNA synthesis and ligation components promotes replication stress.	

While both toxins contribute to the pathogenic process, their interaction is complex and reveals an evolutionary strategy. On the surface, their actions appear antagonistic. VacA is a potent inducer of apoptosis, a protective mechanism that would eliminate a damaged cell. However, CagA actively counteracts this VacA-induced apoptosis. This is achieved through two complementary mechanisms: Tyrosine-phosphorylated CagA intercepts endocytosed VacA, preventing it from reaching its intracellular targets. Unphosphorylated CagA acts at the mitochondrial level to block the apoptotic cascade initiated by VacA. This antagonism is not a contradiction but rather a synergy. By disabling VacA’s cell-killing function, CagA ensures the survival of the host cell. However, the affected cells have accumulated DNA damage, which poses a potential cancer risk (11).

#### 2.1.2 Salmonella Typhi

Salmonella Typhi (S. Typhi) is a pathogen that causes typhoid fever in humans. In addition to causing acute systemic infections, a growing body of epidemiological evidence suggests that this bacterium may play a role in the occurrence and development of specific cancers, especially gallbladder cancer (GC). The chronic carrier state of Salmonella Typhi which is particularly common in patients with gallbladder stones has been confirmed to be an important risk factor for gallbladder cancer (14). Recent studies have shown that compared with non-carriers, chronic carriers of Salmonella Typhi have a significantly increased risk of developing gallbladder cancer, which is 8.47 times that of the latter (15). S. Typhi possesses the ability to directly damage host cell DNA, which is considered one of its most direct carcinogenic mechanisms. This effect is primarily mediated by its unique typhoid toxin.

Typhoid toxin is a unique cell cycle-blocking lethal toxins (CDT), whose distinctive structure enables its delivery into host cells to exert toxic effects (16). Among its components, CdtB possesses the greatest carcinogenic potential. CdtB exhibits DNase I-like activity. Once transported into the host cell nucleus via the typhoid toxin complex, CdtB directly targets chromatin, inducing DSBs (17).

This DNA damage sets off a cascade of events, including the recruitment of the DNA damage sensor complex Mre11-Rad50-Nbs1, which initiates DNA end resection to produce a 3’ overhang. This leads to the accumulation and full activation of the ATM kinase at the site of damage (18). ATM activation results in the phosphorylation of histone H2AX and the activation of several DNA damage checkpoints, including the tumor suppressor p53 and its downstream effector p21, which induces cell cycle arrest in the G1 phase (19). Furthermore, ATM can activate checkpoint kinase 2 (Chk2), and Chk2 in turn inactivates the cell division cycle 25 phosphatase (Cdc25); this process leads to the accumulation of hyperphosphorylated cyclin-dependent kinase 1 (CDK1), ultimately arresting the cell proliferation process at the G2/M phase (20). In some cases, cells exposed to CdtB exhibit markers of cellular senescence, such as 53BP1/cH2AX-positive foci, senescence-associated beta-galactosidase activity, and expansion of promyelocytic nuclear compartments. These pathways allow cells with DNA damage to accumulate genomic instability and mutations through the activation of DNA damage checkpoint responses. The survival of cells with genomic instability is considered a significant factor in cancer development (21).

The Salmonella virulence factor AvrA enters host colonic epithelial cells via a type III secretion system (22). Its core mechanism involves activating the STAT3 signaling pathway and stabilizing β-catenin, which upregulates cytokines such as IL-6 and IL-17. This promotes cell proliferation and fosters an inflammatory microenvironment, thereby accelerating the development of colorectal cancer. AvrA is a key tumor-promoting molecule in infection-associated intestinal carcinogenesis.

CDT can elicit cytotoxic immune responses in cells (23), and the specific domain of typhoid toxin optimizes toxin delivery (14). The ability of cells to survive exposure to CDT is closely linked to the activation of the small GTPase Ras homolog gene family, member A (RhoA). This activation is instrumental in promoting the formation of actin stress fibers. Importantly, this process is not a direct effect of the toxin itself, but rather a cellular response to genotoxic stress, mediated by the activation of ATM kinase. The activation of RhoA, in turn, prevents cell death by activating the p38 pathway and its downstream target, mitogen-activated protein kinase-activated protein kinase 2, thereby prolonging cell survival. Factors that disrupt normal cell cycle progression can potentially lead to the accumulation of mutations. This phenomenon is similar to the effects of genotoxic substances such as ROS and RNS — these substances have been proven to significantly increase the mutation rate in the genome (24). Therefore, in cells with already damaged DNA, the activation of survival responses induced by CdtB may lead to the accumulation of genetic instability within the inflammatory microenvironment. This microenvironment precisely provides ideal conditions for the transformation of pre-cancerous cells into malignant cells, which in turn may promote carcinogenesis in the host.

### 2.2 Virus

Oncogenic virus infection is responsible for 15–20% human cancers (25). There are seven viruses that are confirmed to be oncogenic, including EBV, HTLV-1, HPV, HBV, HCV, KSHV and MCPyV. In contrast to bacteria, viral oncogenesis is characterized expressing oncoproteins. By the integration of viral genetic material into the host genome, viruses directly modulating host cell functions and promoting cancerous transformations through triggering genomic instability, inhibiting apoptosis, and altering cell cycle regulation. The following section briefly summarizes how the oncogenic viruses initiate cancer.

#### 2.2.1 Human papillomavirus

Human Papillomavirus (HPV) is an oncogenic double-stranded circular DNA virus, which mainly infects squamous epithelial cells of skin and mucous membranes. Based on the differences in nucleotide sequences of the L1 gene, HPV can be categorized into more than 200 subtypes. Low-risk HPV (e.g. HPV6, HPV11) can cause benign tumors such as papillomas in the oral cavity and larynx, while high-risk HPV (e.g. HPV16, HPV18) can cause malignant tumors such as cervical cancer and anal cancer. Approximately 70% of all cervical cancers worldwide are caused by HPV 16 and 18 (26).

HPV protein E5, E6 and E7 are major factors in causing oncogenesis. E5 interacts with epidermal growth factor receptors to promote the phosphorylation of AkT and the activation of mTOR, thus inhibiting keratinocyte differentiation and maintaining an undifferentiated state, which facilitate viral replication (27). The high-risk HPV E6 contains a PDZ-binding domain, which helps it target and degrade various cellular proteins that are mediated by the PI3K/Akt signals such as human disc large and membrane-associated guanylate kinase (MAGUK), which helps disrupting cell polarity and signal transduction, activating the PI3K/Akt pathway (28). E7 can bind to and inhibit the retinoblastoma protein (pRB) to release E2F transcription factor, thus eliminating its suppression of the cell cycle and promoting the cell entering the S phase (29). Therefore, the normal regulatory mechanisms is disrupted, and ultimately it results in abnormal cell proliferation.

#### 2.2.2 Epstein-Barr virus

Epstein-Barr virus (Epstein-Barr virus, EBV) is an oncogenic double-stranded DNA virus that primarily infects lymphocytes and oropharyngeal epithelial cells. Globally, EBV infection can be detected in approximately 30%–40% of cases of classical Hodgkin’s lymphoma (cHL). Besides, EBV has a direct causal relationship with the occurrence of several types of other cancers, such as Burkitt lymphoma, nasopharyngeal carcinoma, EBV-associated gastric cancer, and EBV-associated T/NK cell lymphoma (30).

**Figure 2:**
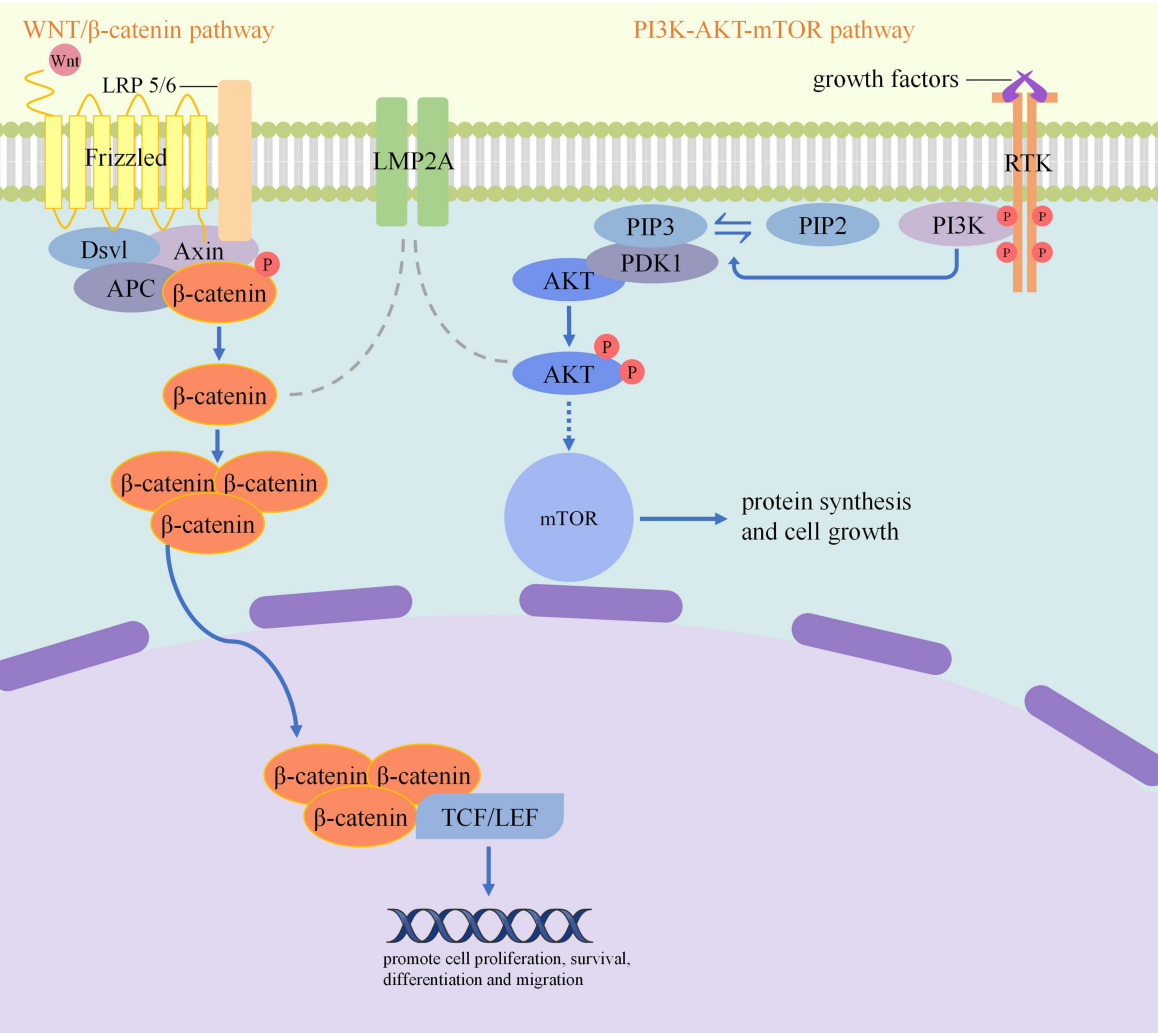
How LMP2A modulates signaling pathways: LMP2A induces AKT phosphorylation and activates the PI3K–AKT pathway, which leads to an anti-apoptotic function that prevents the removal of damaged cells. LMP2A also stabilizes β-catenin, which result in the upregulates downstream genes and increases cell proliferation and promote tumorigenesis.

Latent membrane protein 1 (LMP1) and latent membrane protein 2A (LMP2A),which are expressed by EBV in host cells, are key oncoproteins of the virus. They mainly promote the carcinogenesis process by regulating signaling pathways in host cells. **Figure 2** demonstrates how LMP2A modulates signaling pathways. LMP1 is a potent activator of nuclear factor-κB (NF-κB) signaling. It activates the NF-κB signaling pathway by mimicking the structure and function of TNFR and recruiting IRAK1 and TRAF6. Once activated, this pathway upregulates the expression levels of molecules such as A20 and c-IAP, which ultimately enhances cell survival capacity. In addition, LMP1 can activate the PI3K/Akt pathway, which inactivates the pro-apoptotic proteins Bad and Foxo3a in cells through phosphorylation modification (31). To inhibit apoptosis, LMP1 also activates BCL2 family proteins (32). What’s more, EBV microRNAs targets genes to help cell survival. The intronic regions of the BamHI rightward transcript (BART) of Epstein-Barr virus (EBV) can synthesize BART-microRNA. BART-microRNA targets Bcl-2 interacting mediator of cell death to eradicate its inhibition of the antiapoptotic action of BCL2.

#### 2.2.3 Hepatitis B virus

Hepatitis B virus (HBV) is an oncogenic partially double-stranded DNA virus which primarily infects hepatocytes, especially the parenchymal liver cells. HBV has a direct causal relationship with hepatocellular carcinoma (HCC). It’s estimated that HBV leads to over 50% of HCC cases globally (33).

**Figure 3:**
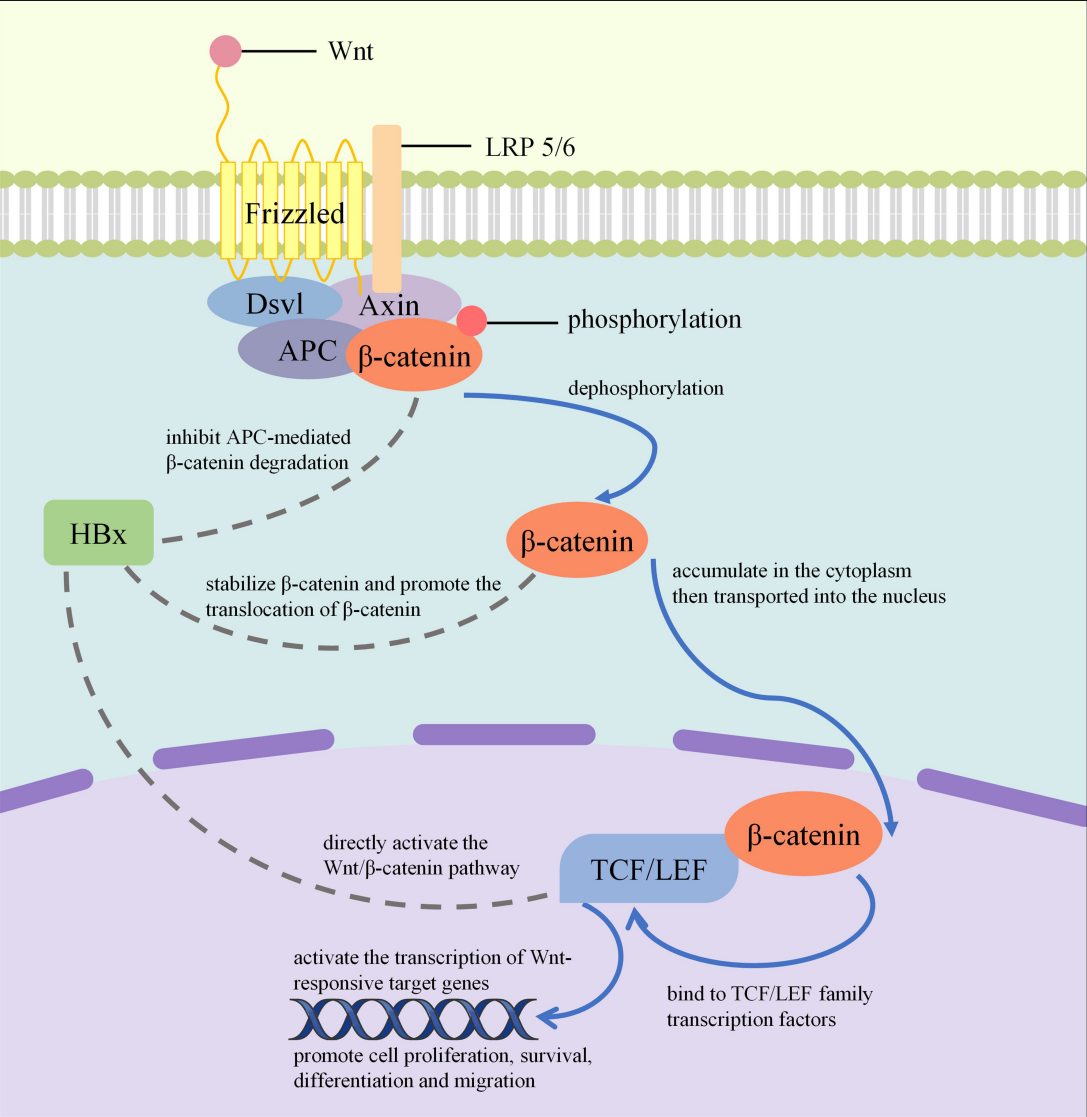
How HBx modulate WNT/β-catenin pathway: Through inhibiting APC-mediated β-catenin degradation, stabilizing β-catenin, promoting the translocation of β-catenin and directly activate the WNT/β-catenin pathway, HBx stimulates abnormal transcription of target genes that drive cell proliferation, which ultimately result in HCC development.

Most HBV breakpoints are close to coding genes. HBV DNA is mainly integrated into exons or regulatory regions, including the telomerase, mixed lineage leukemia protein 4, cyclin 1, Sentrin-specific protease 5 (SENP5) and Rho-associated coiled-coil containing protein kinase 1 genes (34). HBx-LINE1 expression in HBV-related HCCs downregulates microRNA 122, whose role is a potent anti-inflammatory tumor suppressor in liver, leading to the derepression of hundreds of microRNA 122 targeted genes and the disruption of liver homeostasis (35). HBx protein regulates the transcription of viral and host genes and activates multiple cellular signaling pathways which are involved in inflammation and proliferation, such as the Wnt/β-catenin pathway (36), MAPK pathway (37), thus promoting cell proliferation and inhibit apoptosis. **Figure 3** demonstrates how HBx modulate WNT/β-catenin pathway. HBx protein can bind the chromatin of host cells, affecting gene expression and promoting viral replication and cell transformation. Mutations in preS/S proteins can lead to endoplasmic reticulum stress, activating the unfolded protein response, increasing intracellular oxidative stress and DNA damage, thus promoting the transformation of hepatocytes (38).

#### 2.2.4 Human T-lymphotropic virus 1

Human T-lymphotropic virus 1 (HTLV-1) is an oncogenic single-stranded RNA retrovirus which mainly infects CD4 + T cells and integrates into host DNA (39). HTLV-1 has a direct causal relationship with adult T-cell leukemia/lymphoma (ATL) (40). In the oncogenic process of human T-lymphotropic virus type 1 (HTLV-1), two key regulatory proteins, the HTLV-1 transactivator x (Tax) and the HTLV-1 basic leucine zipper factor (HBZ) are encoded by it and play important roles.

Tax is a transactivator of viral RNA and has the capability to induce viral replication which helps increasing genetic instability. Tax leads to the persistent phosphorylation, ubiquitination, and subsequent proteasomal degradation of IκBα, triggering NF-κB activation and inducing the constitutive phosphorylation and activation of the inhibitor of NF-κB kinase (IKK) complex in an aim of sustaining NF-κB activation (41). Tax activates CDK4 through inhibiting its inhibitors including p15, p16, p18 and p19, thus facilitating S phase entry and cell proliferation (42).

HBZ is an antisense transcript of the HTLV-1 provirus and contributes greatly in viral replication and T-cell proliferation. C/EBPα is a vital negative regulator of cell proliferation. HBZ interacts with C/EBPα and diminishes its DNA binding capacity, thereby overcoming the suppressive effect of C/EBPα on cell growth, leading to cell proliferation (43). Besides, through dysregulating the WNT signaling pathway, it promotes migration and proliferation (44).

#### 2.2.5 Hepatitis C virus

Hepatitis C virus (HCV) is an oncogenic single-stranded positive-sense RNA virus. There is a clear causal relationship between HCV infection and hepatocellular carcinoma (HCC) (45). The mechanisms HCV employs to induce HCC include triggering oxidative stress, contributing to hepatic steatosis and deregulating cellular pathways.

HCV core, E1, E2, NS4B, and NS5A proteins can induce the release of calcium ions and hydrogen peroxide in the endoplasmic reticulum (ER), thereby triggering endoplasmic reticulum oxidative stress (46, 47). NS5A also facilitates calcium uptake in the mitochondria and ER, leading to increased production of ROS. In addition, the HCV core protein and NS5A further elevate ROS levels by upregulating cytochrome P450 2E1 and NADPH oxidases (48). Both hydrogen peroxide which is released into the cytoplasm or nucleus and the increase of ROS may lead to direct DNA damage, thus promoting gene mutations and carcinogenesis (49).

HCV proteins activate cell survival and proliferation signaling pathways through various mechanisms. For example, NS5B binds to the tumor suppressor Rb to facilitate proteasomal degradation, releasing E2F to promote the expression of cell cycle-dependent genes, thus advancing the cell cycle and evading the G1/S checkpoint (50). NS5A activates the PI3K/Akt pathway by binding to and inactivating the tumor suppressor phosphatase and tensin homolog (pTEN), which promotes cell proliferation and survival (51). Additionally, the HCV core protein, E2, NS3, and NS5A interact with the RAF/MAPK/ERK pathway to promote cell proliferation (52).

#### 2.2.6 Kaposi sarcoma-associated herpesvirus

Kaposi sarcoma-associated herpesvirus (KSHV) is an oncogenic double-stranded DNA virus that can directly induce Kaposi sarcoma (KS) (53). The mechanisms it employs mainly include promoting cell proliferation and escaping from host immune surveillance.

Oncoproteins of KSHV can subvert pathways controlling cellular proliferation to promote cell proliferation. **Table 2** demonstrates the signaling pathways modulated by oncogenic virus factors of KSHV. The LANA protein encoded by KSHV can stably regulate β-catenin by activating the WNT/β-catenin signaling pathway; this regulatory effect further upregulates the expression levels of downstream target genes (such as CCND1 and MYC), thereby promoting abnormal cell proliferation and ultimately driving tumorigenesis (61). Additionally, the K1 protein of KSHV inhibits apoptosis by activating the AKT signaling pathway, thereby protecting virus-infected cells from premature death and providing favorable conditions for KSHV-induced tumorigenesis (62).

**Table 2:** Signaling pathways modulated by KSHV.

Oncogenic virus factors	Signaling pathways	Mechanisms	References
vGPCR	PI3K–AKT–mTOR	activating AKT phosphorylation	(54)
	Notch	inducing the expression of core Notch receptors and ligands	(55)
LANA	Notch	competitively inhibiting the interaction between ICN and FBXW7	(55)
	WNT/β-catenin	stabilizing β-catenin	(56)
K1	PI3K–AKT–mTOR	activating AKT signaling	(57)
ORF45	PI3K–AKT–mTOR	upregulating mTOR signaling	(58)
kaposin B	MAPK	binding to and activating MK2K	(59)
vFLIP	Notch	inducing the expression of core Notch receptors and ligands	(55)
	NF-κB	associating with IKK complex component	(60)
vIL-6	Notch	inducing the expression of core Notch receptors and ligands	(55)

#### 2.2.7 Merkel cell polyoma virus

Merkel cell polyomavirus (Merkel cell polyomavirus, MCPyV) is an oncogenic double-stranded DNA virus that has a direct causal relationship with Merkel cell carcinoma (63). The mechanisms it employs mainly include promoting cell proliferation and manipulating the host DNA damage response.

The small T oncoprotein of MCPyV can target 4E-BP1 downstream of the PI3K–AKT–mTOR signaling pathway, promoting its phosphorylation. This leads to excessive activation of cap-dependent translation, thereby facilitating cellular transformation (64). By activating the mTOR signaling pathway, MCPyV can promote cell proliferation and survival, thus providing favorable conditions for tumorigenesis.

The large T antigen of MCPyV can activate the host’s DDR. However, at the same time, it can also inhibit the normal function of DDR through multiple mechanisms, leading to genomic instability. For example, the large T antigen of MCPyV can bind to and inhibit key proteins in the DNA, such as ATM and ATR. Thus, it impedes the repair of DNA damage, increases the risk of gene mutations, and promotes the development of tumors (65).

### 2.3 Fungus

Aspergillus is a type of fungus that is widely found in nature, especially in soil, decaying plants, and in the air. Although most Aspergillus species are harmless to humans, certain types such as Aspergillus fumigatus can cause infections, especially in individuals with weakened immune systems. Certain secondary metabolites produced by some species of Aspergillus, such as aflatoxins, possess strong carcinogenic properties, particularly aflatoxin B1(AFB1), which is closely associated with the occurrence of liver cancer (66).

The TP53 R249S mutation is a common mutation type in HCC, especially significant in the context of exposure to AFB1 and co-infection with HBV (67). TP53 is an important tumor suppressor gene responsible for regulating the cell cycle, DNA repair, and apoptosis. The R249S mutation disrupts the normal function of the p53 protein, leading to a loss of DNA repair capacity in cells and suppression of apoptosis, thereby promoting tumor development. HBV infection can induce an increase in cytochrome P450 enzyme activity within hepatocytes, metabolizing AFB1 into the highly active AFB1-8,9-epoxide. The latter forms adducts with DNA, leading to TP53 mutations. Chronic inflammation and hepatocyte necrosis-regeneration caused by HBV infection further increase the risk of AFB1-induced TP53 mutations. Studies have shown that HBV infection and AFB1 exposure have a synergistic effect, significantly increasing the risk of liver cancer. Among individuals infected with HBV, even moderate exposure to AFB1 can increase the risk of liver cancer by 2-fold (68).

Beyond the carcinogenic metabolites produced by some *Aspergillus* species, recent evidence has indicated that the fungi themselves can reside within tumor tissues and actively modulate the tumor microenvironment, as exemplified by *Aspergillus sydowii* in LUAD. It activates the Dectin-1/CARD9 signaling pathway via cell wall β-glucan, inducing macrophages to secrete IL-1β, which subsequently promotes the expansion and activation of myeloid-derived suppressor cells (MDSCs). This process suppresses cytotoxic T cell function and leads to the accumulation of regulatory T cells (Tregs) and PD-1^+^CD8^+^ T cells, thereby shaping an immunosuppressive TME and accelerating tumor progression (69).

Although aflatoxins are primarily linked to liver cancer, studies also suggest a possible association with other cancers such as stomach and colorectal cancer, but the evidence is not yet conclusive. In immunosuppressed patients (such as organ transplant recipients, HIV-infected individuals, or those undergoing chemotherapy), fungi from the genus Aspergillus (such as Aspergillus fumigatus) can cause invasive aspergillosis (70). Chronic inflammation and immunosuppressed states may indirectly increase the risk of cancer. For instance, prolonged inflammatory responses can lead to the production of ROS and RNS, which can cause DNA damage and genomic instability, thereby increasing the likelihood of cancer development. Aspergillus fungi are widely present in the environment and may indirectly affect the risk of cancer by producing other secondary metabolites or causing chronic inflammation (71).

## 3 Pathogens affecting tumor progression

Pathogens influencing tumor progression do not initiate carcinogenesis but modulate tumor development through indirect mechanisms. Bacteria such as Fusobacterium nucleatum regulate signaling pathways, enhance chemoresistance, and suppress anti-tumor immunity. Viruses reshape the tumor microenvironment via immune dysregulation, cytokine secretion, and promotion of angiogenesis. Fungi like Candida albicans affect immune responses and cytokine profiles, facilitating tumor growth and metastasis. These pathogens synergize with existing malignant cells to accelerate tumor progression and impair therapeutic efficacy.

### 3.1 Bacteria

In contrast to direct carcinogens, various commensal or opportunistic pathogenic bacteria can reshape the local ecosystem by driving tumor progression and metastasis by modulating the tumor microenvironment, influencing host immunity, or altering metabolism. A prominent example is Fusobacterium nucleatum. Furthermore, other bacteria like Peptostreptococcus anaerobius have been shown to promote colorectal carcinogenesis and modulate tumor immunity (72). Recent studies also reveal that gut-derived Klebsiella pneumoniae can translocate to the liver and promote hepatocellular carcinoma development in mouse models (73). This section will focus on Fusobacterium nucleatum to elucidate how bacteria act as key ecological modulators of tumor progression and outline their mechanisms of action.

#### 3.1.1 Fusobacterium nucleatum

Fusobacterium nucleatum (F. nucleatum) is an anaerobic, Gram-negative bacterium which is commonly found in human oral cavity and gastrointestinal tract. Characterized by its spindle-shaped morphology, it functions as both a commensal organism and opportunistic pathogen. It has been linked to systemic conditions, including colorectal cancer, through mechanisms like promoting tumorigenesis and metastatic progression. Fusobacterium nucleatum employs various strategies to influence cellular processes and immune responses. The adhesin FadA binds to E-cadherin, activating the β-catenin/WNT signaling pathway to promotes cell proliferation (74). Meanwhile, the bacterial endotoxin lipopolysaccharide (LPS) activates Toll-like receptor 4 (TLR4), triggering the upregulation of miR21. This activates the rat sarcoma–mitogen-activated protein kinase cascade, enhancing cell proliferation (75, 76). Additionally, LPS interactions with TLR4 upregulate baculoviral IAP repeat containing 3, which inhibits apoptosis by directly inhibiting the caspase cascade. Thereby the cell resistance against cytotoxic drugs is increased (77). These interactions also downregulate the expression of miR18a and miR4802, which are associated with autophagy elements Unc-51 Like Kinase 1 and ATG7, contributing to increased autophagy and subsequently enhancing cell resistance to therapy (78, 79). F. nucleatum further inhibits apoptosis by upregulating the expression of anoctamin-1 in a TLR4-dependent manner, contributing to chemoresistance (80). F. nucleatum produces the trimeric autotransporter adhesin CbpF, which specifically binds to Carcinoembryonic Antigen-related Cell Adhesion Molecule 1(CEACAM1) on TILs and NK cells, thereby suppressing the antitumor activity of TILs and NK cells by activating the human inhibitory checkpoint receptor CEACAM1.And the non-lectin domain of Fibroblast Activation Protein Alpha inhibits the anti-tumor activity of tumor-infiltrating lymphocytes (TILs) and natural killer (NK) cells by activating the human inhibitory receptor T cell immune receptor with Ig and ITIM domains checkpoint (81).

### 3.2 Virus

In addition to 7 oncogenic viruses, HIV, despite unable to directly initiate cancer, also contributes to cancer progression. Unlike the initial viral events that trigger oncogenesis, the mechanisms promoting tumor progression mainly rely on the post-transformation roles of oncogenic viruses, including their effects on chronic inflammation, immune evasion, metabolic reprogramming, and modulation tumor microenvironment. This section will examine how persistent viral infection affects cancer progression, especially focusing on HIV.

#### 3.2.1 Human papillomaviruses

**Figure 4:**
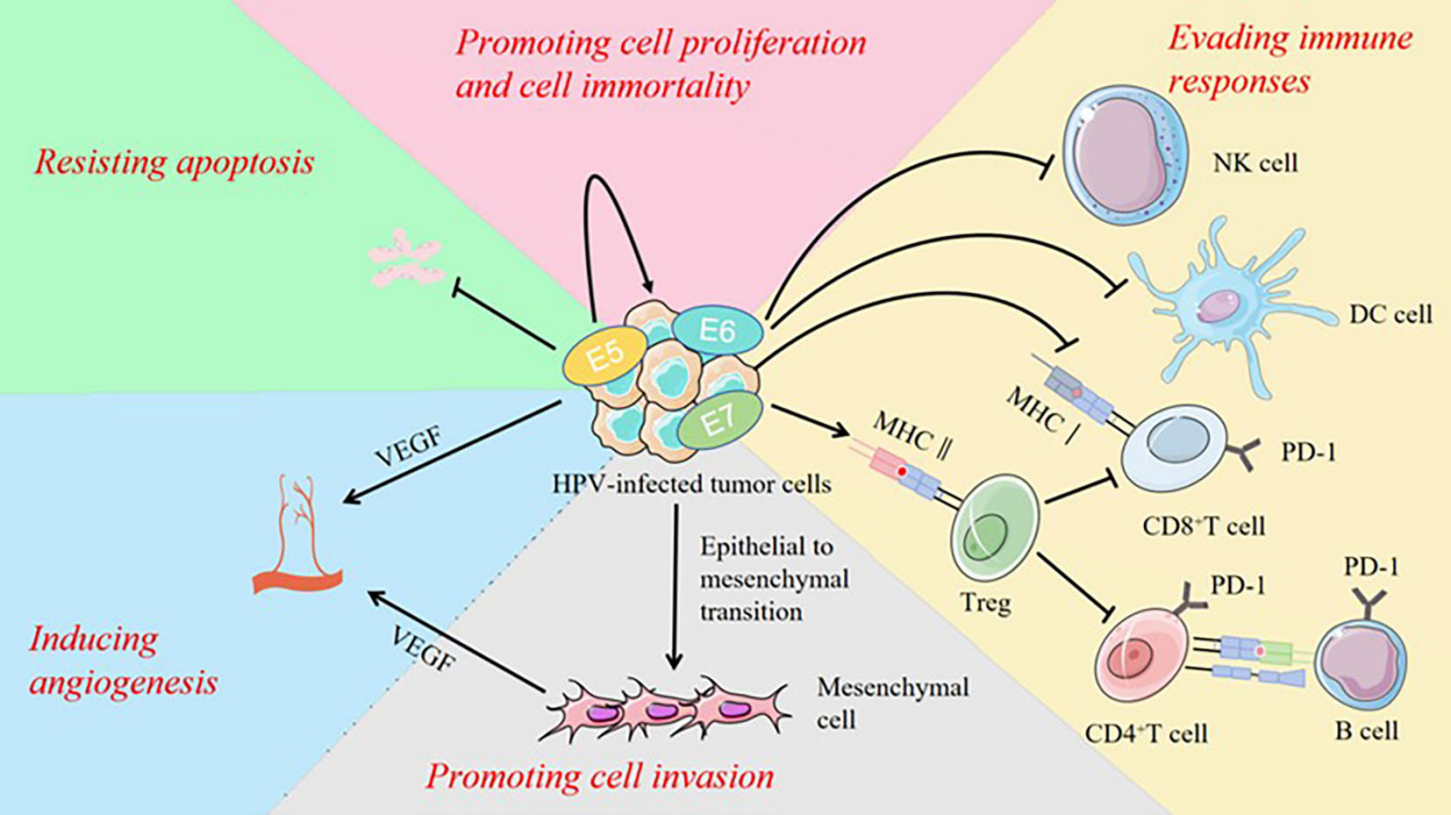
High risk HPVs drive cancer oncogenesis and progression through multiple mechanisms, including immune evasion, promotion of cell proliferation, resistance to apoptosis, induction of cellular immortality, angiogenesis, and enhancement of cell invasion.

The HPV genome is structured into three distinct regions: the non-coding region, which influences replication and transcription, the early region, which codes for the E1, E2, E4, E5, E6, and E7 proteins and the late region, which encodes the L1 and L2 proteins that serve as the viral capsid proteins (82). **Figure 4** summarizes the main mechanisms high risk HPV employs to affect tumor progression.

The Scribble, Crumbs, and Par complexes—all with a Postsynaptic density 95,Discs large and Zonula occludens-1 (PDZ) motif—are critical for maintaining cellular polarity (83). Membrane-associated guanylate kinase 1, a tumor suppressor in cancer progression, is targeted and degraded by E6, disrupting cell adhesion to promote invasion and metastasis. E6 disrupts PDZ domain–containing polarity regulators, including Scribble, Crumbs, and Par complexes, leading to loss of cell–cell adhesion and activation of pro-invasive signaling pathways. This polarity dysregulation promotes epithelial-to-mesenchymal transition (EMT) and enhances metastatic potential (84).

ntributes to progression through cooperative effects with E6, including upregulation of miR-18a and suppression of Hippo/STK4 signaling, thereby enhancing proliferative capacity. E7 further promotes growth by increasing Myelocytomatosis oncogene (c-Myc)c-Myc and BCL-2-associated X protein levels.

#### 3.2.2 Epstein-Barr virus

EBV has two distinct life cycle phases—latency and lysis—both involved in epithelial tumor oncogenesis. In the duration of latency, EBV expresses specific genes such as EBV nuclear antigen 1 (EBNA1), LMP1, and LMP2A. Through disrupting cellular circle, evading immune surveillance, these viral proteins can drive malignant transform. Lytic replication is essential in viral transmission as it enhances malignancy via releasing cytokine, inducing angiogenesis, and modifying extracellular matrix (85). Both latent infection and lytic replication of EBV contribute to epithelial tumor development. Studies show that EBNA1—a key latent viral protein universally expressed in all EBV-associated cancers—collaborates with the transcription factor CCAAT enhancer binding protein beta at the KDM5B promoter to enhance its transcription. Additionally, via its DNA-binding domain, EBNA1 binds to host gene promoters, notably upregulating c-Jun and ATF2 which is critical for nasopharyngeal carcinoma (NPC) development and metastasis. It also mediates tethering of EBV and host genomes to regulate enhancers, promoting NPC progression (85).

#### 3.2.3 Hepatitis B virus

HCC develops within a highly complex TME that is shaped by chronic inflammation and fibrosis, setting it apart from many other cancer types. HBV infection plays a central role in initiating and sustaining this pathogenic milieu by triggering persistent inflammatory and fibrotic processes. Concurrently, HBV infection drives the infiltration of leukocytes and the activation of tissue-resident immune cells and fibroblasts, further reshaping the TME. This dynamic and self-reinforcing microenvironment supports HCC development from promotion,(where fibrotic niches foster the growth of premalignant lesions) to progression(where immune evasion and stromal interactions enable invasive tumor growth). The interplay of these factors underscores the unique complexity of the liver TME and its critical role in HCC pathogenesis (86).

Hepatic tumor cells also communicate with non-tumor stroma, which comprises extracellular matrix (ECM) components, non-malignant fibroblasts, immune cells, and endothelial cells—collectively forming the peri-tumoral microenvironment. Previous studies indicate that cross-talk between tumor cells and this abnormal microenvironment (including inflammatory cytokines, upregulated growth factors, ECM, and chemokines) promotes angiogenesis. Activated hepatic stellate cells (HSCs) are key drivers of liver fibrosis and cirrhosis. As a critical component of the HCC cellular microenvironment, HSCs are responsible for hepatic collagen synthesis. Based on liver damage, activated HSCs gather in the ECM, thus inducing hepatic fibrosis. What’s more, paracrine interactions between activated HSCs and hepatocytes influence HCC proliferation and metastasis (87).

Additionally, multiple immune mechanisms are dysregulated during HCC progression (88). HCC develops in a chronically inflamed liver microenvironment, which drives the production of inhibitory cytokines such as IL-10 and TGF-β. These cytokines lead to the decrease of anti-tumor immune responses while promoting tumor growth (89). Moreover, immune cells in HCC frequently upregulate checkpoint inhibitor receptors like cytotoxic T lymphocyte-associated antigen-4 and PD-1.The increased expression of these receptors impairs immune cell activation, thus preventing effective tumor clearance (87).

#### 3.2.4 Hepatitis C virus

HCC development in HCV infection arises from a multifactorial interplay of viral, host, and environmental determinants. Chronic inflammation drives fibrosis and cirrhosis, linked to growth factor secretion, immune suppression, and angiogenesis—closely associated with HCC development. HCV directly contributes to fibrogenesis beyond chronic inflammation. n HCV-associated HCC, HCV activates Wnt/β-catenin signaling in infected hepatocytes *in vitro*, alongside enriched activating catenin beta 1 mutations (90). It also induces TGF-β secretion via virus-host interactions, ER stress, and ROS production; this TGF-β promotes fibrogenesis by activating hepatic stellate cells (91). Notably, TGF-β acts stage-specifically: initially suppressing tumorigenesis, but later enhancing progression via proliferation, angiogenesis, and EMT (92). High immune infiltration is also usually found in HCV-associated HCC, but most cells are exhausted T cells. T cell exhaustion in HCV-infected patients is largely antigen-specific (93–95).

#### 3.2.5 Kaposi sarcoma-associated herpesvirus

**Figure 5:**
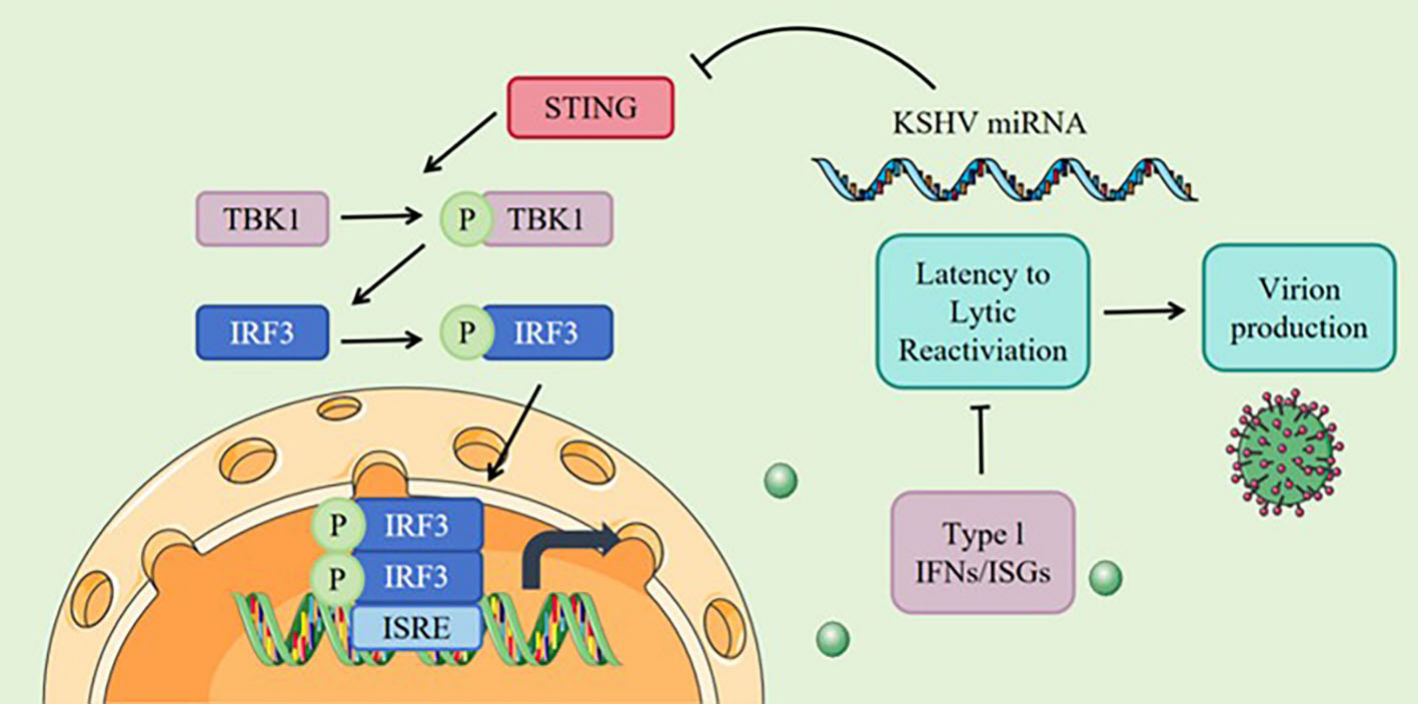
KSHV immune evasion mechanism. The exogenous delivery of KSHV miRNAs resulted in reduced STING expression and weakened cGAS/STING signaling when cells were stimulated with STING agonists. In contrast, genetic deletion of these KSHV miRNAs restored the expression of STING and interferon-stimulated genes in KSHV latently infected cell lines, which in turn delayed KSHV lytic reactivation and decreased the expression of KSHV lytic genes.

The roles of specific KSHV genes expressed during latency or the lytic cycle differ across tumor types. **Table 3** shows the major expressed viral genes in KSHV-related cancers. KSHV adopts multiple tactics to escape host immune surveillance and sustain lifelong latency. **Figure 5** summarizes the immune evasion mechanisms KSHV employs. A key target of this evasion is the cyclic GMP–AMP synthase–stimulator of interferon genes (cGAS–STING) pathway, which serves as a principal sensor of viral DNA and restricts KSHV replication during reactivation. Recent evidence indicates that three viral microRNAs—miR-K12-6-3p, miR-K12-7-3p, and miR-K12-11-3p—can directly interact with the mRNA of STING1, thereby reducing its translation and dampening downstream interferon signaling. When these miRNAs were experimentally introduced into host cells, STING levels decreased, accompanied by a marked suppression of cGAS–STING pathway activation following stimulation with STING agonists. In contrast, deletion of these miRNAs from latent KSHV genomes restored STING expression and interferon-stimulated gene activity, consequently delaying viral reactivation and diminishing the expression of lytic-phase genes. Collectively, these results delineate a novel immune evasion mechanism in which KSHV miRNAs suppress STING-mediated antiviral signaling—representing the first known instance of viral miRNAs directly targeting this pathway. Therapeutic approaches under exploration include inhibition of viral gene functions, restoration of perturbed host signaling cascades, modulation of cytokine production, and broader immunotherapeutic interventions (98).

**Table 3:** KSHV latent gene expressed in virus-associated tumors. This table shows the major expressed viral genes in KSHV-related cancers.

Cancer	The major expressed viral genes	References
Kaposi sarcoma (KS)	LANA and vGPCR	(96)
KSHV-associated MCD (K-MCD).	huIL-6, huIL-10, and vIL-6	(97)
Primary Effusion Lymphoma (PEL)	LANA	(97)

#### 3.2.6 Merkel cell polyoma virus

The viral DNA found within merkel cell carcinomas is often integrated into the host cell’s genome. This integration happens at various, albeit more frequently at chromosome 5, locations in the genome; it may occur as either one copy or a series of multiple copies (99). The integration typically leads to tumor-specific truncation mutations that maintain the expression of sT and LT truncation (LTT) mutants, which conserve the N-terminal RB-binding LXCXE motif critical for inhibiting RB’s tumor suppressive role (100–104). LTT and sT proteins, expressed from the integrated viral genome, act as the primary oncoproteins driving continued tumor cell proliferation and viability (64, 105, 106).

#### 3.2.7 HIV

HIV consists of two strands of RNA, fifteen types of viral proteins, along with a small number of proteins derived from the last host cell it infected. All these components are enclosed by a lipid bilayer membrane. Collectively, these molecules enable the virus to infect immune system cells and compel them to produce new copies of the virus. In comparison to the general public, individuals infected with HIV currently have approximately a 500 - fold increased likelihood of being diagnosed with Kaposi sarcoma, a 12 - fold higher probability of being diagnosed with non - Hodgkin lymphoma, and among women, a 3 - fold greater chance of being diagnosed with cervical cancer (107). Moreover, HIV - infected people are at a heightened risk of several other cancer types which are collectively referred to as “non - AIDS - defining cancers” (107).

The development of lymphomas in HIV-infected patients is driven by mixed factors, including immune dysregulation, loss of control over oncogenic viruses, genetic alterations, and impaired T-cell immunosurveillance. HIV contributes to lymphomagenesis indirectly by creating a tumor-promoting microenvironment. This is achieved through chronic immune activation, particularly B-cell hyperactivation, which is a hallmark of HIV infection (108).

Another key mechanism involves HIV-encoded proteins. For example, the Tat (transactivator of transcription) protein plays a critical role in HIV transcription and disease progression. Studies have shown that approximately 30% of mice transgenic for the HIV Tat protein develop B-cell lymphomas (109), highlighting its potential oncogenic role. Tat in lymphoid tissues can enter B lymphocytes and disrupt cellular regulatory mechanisms, deregulating the pRb2/p130 tumor suppressor protein (110). Additionally, Tat promotes lymphomagenesis by enhancing the production of growth-promoting cytokines such as IL-6 and IL-10 (111, 112), upregulating DNA repair enzymes like β-polymerase (113), and stimulating angiogenesis (114). These activities collectively create a microenvironment conducive to B-cell proliferation and malignant transformation.

While HIV genomic sequences are rarely found within lymphoma cells, tumor-associated macrophages have been shown to harbor retroviral insertions (115). These infected macrophages secrete inflammatory cytokines that further drive B-cell proliferation, eventually leading to monoclonal expansion and lymphoma development. Furthermore, HIV viral proteins can indirectly promote B-cell proliferation by inducing alterations in T-cell genes, thereby disrupting immune regulation (108).

### 3.3 Fungus

#### 3.3.1 Candida

Candida is a common opportunistic pathogenic fungus, which is widely found in the environment and in the human body. It may cause various infections when the immune system is compromised. Currently, there is no direct evidence to suggest that Candida (such as Candida albicans) directly causes cancer, but it may indirectly promote cancer through various mechanisms.

Candida albicans can produce nitrosamines, a substance known to alter the proliferation of oral cancer cells. Candida albicans infection can also affect the tumor microenvironment by modulating the host’s immune response (116). During the infection process, Candida albicans activate dendritic cells and macrophages, inducing the release of specific cytokines such as tumor necrosis factor α (TNF-α), IL-6, IL-10, and IL-18. These cytokines play a dual role in inflammatory responses, potentially inhibiting tumor development and possibly promoting the growth and metastasis of tumor cells. In certain cases, TNF-α can promote cell survival and proliferation by activating the NF-κB signaling pathway, thereby exacerbating tumor growth and metastasis. The overexpression of IL-18 can suppress the immune system’s attack on tumor cells, thus facilitating tumor immune escape. This cytokine is considered to play a key role in regulating Th1-type immune responses. IL-10 is an anti-inflammatory cytokine that can inhibit effective anti-tumor immune responses. Candida albicans may enable tumor cells to evade immune surveillance by promoting the production of IL-10.

Furthermore, Candida albicans promote the adhesion of tumor cells to epithelial cells, creating conditions for tumor growth and spread. In mouse models of breast cancer and melanoma, the interaction between fungi (such as Candida albicans) and bacteria can lead to differences in the tumor’s response to radiation therapy. This suggests that Candida may indirectly affect the tumor’s sensitivity to treatment through interactions with other microbes, but the specific mechanisms of action remain to be studied (117). Streptococcus mutans can form Biofilms, which protect cancer cells and enhance drug resistance, are formed by Streptococcus mutans on medical equipment and tissue surfaces, increasing the risk of infection and promoting tumor development (118).

#### 3.3.2 Malassezia

Malassezia is a genus of conditional pathogenic fungi widely present on human and animal skin surfaces. Characterized by strict lipophilicity, it frequently colonizes areas with abundant sebum secretion and can, under specific conditions, induce skin diseases or even participate in tumor progression.

The Malassezia genus plays a critical oncogenic role in the development of pancreatic ductal adenocarcinoma (PDA) through specific colonization and signaling pathway activation (119). Malassezia can migrate from the intestinal tract to the pancreas via the sphincter of Oddi and demonstrates specific enrichment in PDA tumor tissues in both mouse models and humans, with its abundance significantly higher compared to normal pancreatic tissue. This effect is not observed with other fungi such as Candida, Saccharomyces cerevisiae, or Aspergillus. Its tumor-promoting function relies on the activation of the MBL-C3 complement pathway: glycoproteins on the Malassezia cell wall bind to host mannose-binding lectin (MBL), initiating the lectin complement pathway. This leads to the generation of C3 convertase and the production of C3a. Upon binding of C3a to the C3a receptor on tumor cell surfaces, it directly promotes tumor cell proliferation, migration, and invasion, while simultaneously disrupting adaptive immune responses to foster a pro-tumorigenic microenvironment.

## 4 Discussion

In this review, we summarized the oncogenic pathogens, with particular emphasis on comparing the pathogens that directly cause tumors with the pathogens that affect tumor progression. Generally speaking, direct carcinogenic mechanisms include affecting tumor suppressor pathways, initiating host cell DNA damage and regulating immune responses, while indirect carcinogenic mechanisms involve modulating TME and escaping immune surveillance.

Despite not mentioned in preceding text, researching carcinogenic microorganisms is pivotal. With rapid advances in microbiome and virome profiling, a variety of therapeutic strategies that either target specific microbial components or exploit relevant microorganisms for cancer treatment can be envisioned. For instance, genetically engineered S. Typhi strains have displayed the ability to preferentially accumulate in tumors and stimulate anti-tumor immunity, which may pave the way for bacterial-mediated cancer therapy (120). When it comes to oncogenic viruses, prophylactic HPV vaccines have dramatically reduced the incidence of cervical and other HPV-associated cancers, highlighting the success of infection-targeted cancer prevention. As for fungi, specific fungal DNA signatures have been correlated with tumor subtype, immune infiltration, and survival outcomes in cancers such as colorectal and pancreatic cancer, making it a promising source of new biomarkers (121).

Nevertheless, several formidable challenges still hinder the full understanding and clinical translation of microbe-related oncogenesis. First and foremost, most present studies rely on correlative data derived from animal models or *in vitro* experiments, lacking causal validation in humans. Most current evidence comes from cross-sectional microbiome studies, which cannot distinguish microbial drivers of tumorigenesis from microbial passengers that emerge secondary to tumor progression. Although many studies have suggested that intratumoral microbiome can affect tumor progression, we still cannot confirm whether intratumoral microbiome development is shaped by tumor progression. Second, methodological inconsistencies in microbiome profiling (such as sample handling and sequencing platforms) reduce data reproducibility and cross-study comparability. The ultra-low microbial biomass of tumors further increases the risk of contamination, and the field still lacks standardized, validated protocols for tissue processing, decontamination, viability assessment, and intratumoral microbe quantifications. Furthermore, the cancer microbiome field remains to be not fully explored. Studies are limited by several factors such as sample size and DNA extraction efficacy. Amplicon- based sequencing approaches are hindered by variations in marker gene copy numbers and the amplified region used by different studies (121). In addition, many proposed mechanisms by which microbes influence host epigenetics, metabolism, or immune signaling remain hypothetical due to the lack of functional validation and spatially resolved data.

There are several directions that future researches can focus on. First, integrated multi-omics approaches including metagenomics, metatranscriptiomics, metabolomics, and single-cell or spatial transcriptomics are needed to precisely map microbe-host interactions and identify microbial metabolites or pathways that modulate oncogenic signaling. In addition to animal models, future researches can leverage organoid co-culture systems, humanized mouse systems, and spatial imaging techniques to directly demonstrate the role of microbes in driving tumorigenesis, such as gene knock - out, colonization experiments and metabolite perturbation studies will be particularly important. Interdisciplinary quantitative approaches will also be required to define causal relationships between intratumoral microbes and tumor evolution. Second, targeted microbiome engineering such as precision probiotics, engineered bacteria with tumor-tropic behavior, and programmable bacteriophage may enable selective modulation of tumor-associated microbes to enhance immunotherapy response or reduce cancer risk. Additionally, linking intratumoral microbial differences between tissue of healthy and high-risk individuals to tumor development may enable establishing more effective cancer prevention and diagnosis methods. Prospective human studies focusing on pre-cancerous lesions will be critical for identifying early microbial signatures of malignant transformation.

Overall, decoding the relationship between tumor and microbiomes represents a rapidly evolving frontier in precision oncology. Continued methodological refinement, longitudinal human studies, and rigorous functional validation will be essential to translate microbial signatures from intriguing observations into actionable diagnostic, preventive, and therapeutic tools.

## Glossary

TME: tumor microenvironment
H.p: Helicobacter pylori
ROS: reactive oxygen species
RNS: reactive nitrogen species
DSB: double-strand breaks
DDR: DNA Damage Response
VacA: the vacuolating cytotoxin A
CagA: the cytotoxin-associated gene A
S. Typhi: Salmonella Typhi
GC: gallbladder cancer
CDT: cell cycle-blocking lethal toxins
RhoA: Ras homolog gene family, member A
HPV: Human Papillomavirus
pRB: the retinoblastoma protein
EBV: Epstein-Barr virus
LMP1: Latent membrane protein 1
LMP2A: Latent membrane protein 2
NF-κB: nuclear factor-κB
BART: the BamH rightward transcript
EBER: Epstein–Barr small encoded RNA
PKR: double-stranded RNA-dependent protein kinase
HBV: Hepatitis B virus
HCC: hepatocellular carcinoma
HTLV-1: Human T-lymphotropic virus 1
ATL: adult T-cell leukemia/lymphoma
Tax: HTLV-1 transactivator x
HBZ: HTLV-1 basic leucine zipper factor
IKK: the inhibitor of NF-κB kinase
HCV: Hepatitis C virus
KSHV: Kaposi sarcoma-associated herpesvirus
MCPyV: Merkel cell polyoma virus
AFB1: aflatoxin B1
F. nucleatum: Fusobacterium nucleatum
LPS: lipopolysaccharide
TLR4: Toll-like receptor 4
CEACAM1: Carcinoembryonic Antigen-related Cell Adhesion Molecule 1
TILs: tumor-infiltrating lymphocytes
PDZ: ostsynaptic density 95,Discs large and Zonula occludens-1
EMT: epithelial-to-mesenchymal transition
ACTN4: alpha-actinin-4
CC: cervical cancer
c-Myc: Myelocytomatosis oncogene
TIMP-2: tissue inhibitor of metalloproteinase-2
EBNA1: EBV nuclear antigen 1
H3K27me3: H3 Lysine 4 trimethylation
NPC: nasopharyngeal carcinoma
ECM: extracellular matrix
HSCs: hepatic stellate cells
DAMP: damage-associated molecular pattern
IL-33: interleukin 33
cGAS/STING: cyclic GMP-AMP synthase/stimulator of interferon gene
LTT: LT truncation
TNF-α: tumor necrosis factor α

## References

[1] Bruno PS Biggers P Nuru N Versaci N Chirila MI Darie CC . Small biological fighters against cancer: viruses, bacteria, archaea, fungi, protozoa, and microalgae. Biomedicines. (2025) 13(3). doi: 10.3390/biomedicines13030665, PMID: 40149641 PMC11940145

[2] Zhang S Huang J Jiang Z Tong H Ma X Liu Y . Tumor microbiome: roles in tumor initiation, progression, and therapy. Mol BioMed. (2025) 6:9. doi: 10.1186/s43556-025-00248-9, PMID: 39921821 PMC11807048

[3] Fu K Cheung AHK Wong CC Liu W Zhou Y Wang F . Streptococcus anginosus promotes gastric inflammation, atrophy, and tumorigenesis in mice. Cell. (2024) 187:882–896.e17. doi: 10.1016/j.cell.2024.01.004, PMID: 38295787

[4] Moss SF . The clinical evidence linking helicobacter pylori to gastric cancer. Cell Mol Gastroenterol Hepatol. (2017) 3:183–91. doi: 10.1016/j.jcmgh.2016.12.001, PMID: 28275685 PMC5331857

[5] de Martel C Georges D Bray F Ferlay J Clifford GM . Global burden of cancer attributable to infections in 2018: a worldwide incidence analysis. Lancet Glob Health. (2020) 8:e180–90. doi: 10.1016/S2214-109X(19)30488-7, PMID: 31862245

[6] Butcher LD den Hartog G Ernst PB Crowe SE . Oxidative stress resulting from helicobacter pylori infection contributes to gastric carcinogenesis. Cell Mol Gastroenterol Hepatol. (2017) 3:316–22. doi: 10.1016/j.jcmgh.2017.02.002, PMID: 28462373 PMC5404027

[7] Kalisperati P Spanou E Pateras IS Korkolopoulou P Varvarigou A Karavokyros I . Damage, helicobacter pylori and gastric tumorigenesis. Front Genet. (2017) 8:20. doi: 10.3389/fgene.2017.00020, PMID: 28289428 PMC5326759

[8] Szabò I Brutsche S Tombola F Moschioni M Satin B Telford JL . Formation of anion-selective channels in the cell plasma membrane by the toxin VacA of Helicobacter pylori is required for its biological activity. EMBO J. (1999) 18:5517–27. doi: 10.1093/emboj/18.20.5517, PMID: 10523296 PMC1171620

[9] Akazawa Y Isomoto H Matsushima K Kanda T Minami H Yamaghchi N . Endoplasmic reticulum stress contributes to Helicobacter pylori VacA-induced apoptosis. PloS One. (2013) 8:e82322. doi: 10.1371/journal.pone.0082322, PMID: 24349255 PMC3862672

[10] Zhu P Xue J Zhang ZJ Jia YP Tong YN Han D . Helicobacter pylori VacA induces autophagic cell death in gastric epithelial cells via the endoplasmic reticulum stress pathway. Cell Death Dis. (2017) 8:3207. doi: 10.1038/s41419-017-0011-x, PMID: 29238039 PMC5870595

[11] Kontizas E Tastsoglou S Karamitros T Karayiannis Y Kollia P Hatzigeorgiou AG . Impact of helicobacter pylori infection and its major virulence factor cagA on DNA damage repair. Microorganisms. (2020) 8(12). doi: 10.3390/microorganisms8122007, PMID: 33339161 PMC7765595

[12] Xie C Li N Wang H He C Hu Y Peng C . Inhibition of autophagy aggravates DNA damage response and gastric tumorigenesis via Rad51 ubiquitination in response to H. pylori infection. Gut Microbes. (2020) 11:1567–89. doi: 10.1080/19490976.2020.1774311, PMID: 32588736 PMC7524160

[13] Santos JC Ribeiro ML . Epigenetic regulation of DNA repair machinery in Helicobacter pylori-induced gastric carcinogenesis. World J Gastroenterol. (2015) 21:9021–37. doi: 10.3748/wjg.v21.i30.9021, PMID: 26290630 PMC4533035

[14] Di Domenico EG Cavallo I Pontone M Toma L Ensoli F . Biofilm producing salmonella typhi: chronic colonization and development of gallbladder cancer. Int J Mol Sci. (2017) 18(9). doi: 10.3390/ijms18091887, PMID: 28858232 PMC5618536

[15] Jahan F Chinni SV Samuggam S Reddy LV Solayappan M Su Yin L . The complex mechanism of the salmonella typhi biofilm formation that facilitates pathogenicity: A review. Int J Mol Sci. (2022) 23(12). doi: 10.3390/ijms23126462, PMID: 35742906 PMC9223757

[16] Chemello AJ Fowler CC . Alternate typhoid toxin assembly evolved independently in the two Salmonella species. mBio. (2024) 15:e0340323. doi: 10.1128/mbio.03403-23, PMID: 38501873 PMC11005416

[17] Péré-Védrenne C Prochazkova-Carlotti M Rousseau B He W Chambonnier L Sifré E . The cytolethal distending toxin subunit cdtB of helicobacter hepaticus promotes senescence and endoreplication in xenograft mouse models of hepatic and intestinal cell lines. Front Cell Infect Microbiol. (2017) 7:268. doi: 10.3389/fcimb.2017.00268, PMID: 28713773 PMC5491915

[18] Di Domenico EG Romano E Del Porto P Ascenzioni F . Multifunctional role of ATM/Tel1 kinase in genome stability: from the DNA damage response to telomere maintenance. BioMed Res Int. (2014) 2014:787404. doi: 10.1155/2014/787404, PMID: 25247188 PMC4163350

[19] Grasso F Frisan T . Bacterial genotoxins: merging the DNA damage response into infection biology. Biomolecules. (2015) 5:1762–82. doi: 10.3390/biom5031762, PMID: 26270677 PMC4598774

[20] Cuevas-Ramos G Petit CR Marcq I Boury M Oswald E Nougayrède JP . Escherichia coli induces DNA damage *in vivo* and triggers genomic instability in mammalian cells. Proc Natl Acad Sci U.S.A. (2010) 107:11537–42. doi: 10.1073/pnas.1001261107, PMID: 20534522 PMC2895108

[21] Tubbs A Nussenzweig A . Endogenous DNA damage as a source of genomic instability in cancer. Cell. (2017) 168:644–56. doi: 10.1016/j.cell.2017.01.002, PMID: 28187286 PMC6591730

[22] Lu R Wu S Zhang YG Xia Y Zhou Z Kato I . Salmonella protein avrA activates the STAT3 signaling pathway in colon cancer. Neoplasia. (2016) 18:307–16. doi: 10.1016/j.neo.2016.04.001, PMID: 27237322 PMC4887618

[23] Faïs T Delmas J Serres A Bonnet R Dalmasso G . Impact of CDT toxin on human diseases. Toxins (Basel). (2016) 8(7). doi: 10.3390/toxins8070220, PMID: 27429000 PMC4963852

[24] Grivennikov SI Greten FR Karin M . Immunity, inflammation, and cancer. Cell. (2010) 140:883–99. doi: 10.1016/j.cell.2010.01.025, PMID: 20303878 PMC2866629

[25] zur Hausen H de Villiers EM . Cancer “causation” by infections--individual contributions and synergistic networks. Semin Oncol. (2014) 41:860–75. doi: 10.1053/j.seminoncol.2014.10.003, PMID: 25499643

[26] Castellsagué X . Natural history and epidemiology of HPV infection and cervical cancer. Gynecol Oncol. (2008) 110:S4–7. doi: 10.1016/j.ygyno.2008.07.045, PMID: 18760711

[27] Zhang L Wu J Ling MT Zhao L Zhao K-N . The role of the PI3K/Akt/mTOR signalling pathway in human cancers induced by infection with human papillomaviruses. Mol Cancer. (2015) 14(1). doi: 10.1186/s12943-015-0361-x, PMID: 26022660 PMC4498560

[28] Bhattacharjee R Das SS Biswal SS Nath A Das D Basu A . Mechanistic role of HPV-associated early proteins in cervical cancer: Molecular pathways and targeted therapeutic strategies. Crit Rev Oncol Hematol. (2022) 174:103675. doi: 10.1016/j.critrevonc.2022.103675, PMID: 35381343

[29] Chen J Deng Y Ao L Song Y Xu Y Wang CC . The high-risk HPV oncogene E7 upregulates miR-182 expression through the TGF-β/Smad pathway in cervical cancer. Cancer Lett. (2019) 460:75–85. doi: 10.1016/j.canlet.2019.06.015, PMID: 31247272

[30] Tsao SW Tsang CM Lo KW . Epstein–Barr virus infection and nasopharyngeal carcinoma. Philos Trans R Soc B: Biol Sci. (2017) 372(1732). doi: 10.1098/rstb.2016.0270, PMID: 28893937 PMC5597737

[31] Huang J Zhang X Nie X Zhang X Wang Y Huang L . Assembly and activation of EBV latent membrane protein 1. Cell. (2024) 187:4996–5009.e14. doi: 10.1016/j.cell.2024.06.021, PMID: 38996527

[32] Dawson CW Port RJ Young LS . The role of the EBV-encoded latent membrane proteins LMP1 and LMP2 in the pathogenesis of nasopharyngeal carcinoma (NPC). Semin Cancer Biol. (2012) 22:144–53. doi: 10.1016/j.semcancer.2012.01.004, PMID: 22249143

[33] Venook AP Papandreou C Furuse J de Guevara LL . The incidence and epidemiology of hepatocellular carcinoma: a global and regional perspective. Oncologist. (2010) 15 Suppl 4:5–13. doi: 10.1634/theoncologist.2010-S4-05, PMID: 21115576

[34] Sung WK Zheng H Li S Chen R Liu X Li Y . Genome-wide survey of recurrent HBV integration in hepatocellular carcinoma. Nat Genet. (2012) 44:765–9. doi: 10.1038/ng.2295, PMID: 22634754

[35] Liang HW Wang N Wang Y Wang F Fu Z Yan X . Hepatitis B virus-human chimeric transcript HBx-LINE1 promotes hepatic injury via sequestering cellular microRNA-122. J Hepatol. (2016) 64:278–91. doi: 10.1016/j.jhep.2015.09.013, PMID: 26409216

[36] Levrero M Zucman-Rossi J . Mechanisms of HBV-induced hepatocellular carcinoma. J Hepatol. (2016) 64:S84–s101. doi: 10.1016/j.jhep.2016.02.021, PMID: 27084040

[37] Lei Y Xu X Liu H Chen L Zhou H Jiang J . HBx induces hepatocellular carcinogenesis through ARRB1-mediated autophagy to drive the G(1)/S cycle. Autophagy. (2021) 17:4423–41. doi: 10.1080/15548627.2021.1917948, PMID: 33866937 PMC8726737

[38] Schlüter V Meyer M Hofschneider PH Koshy R Caselmann WH . Integrated hepatitis B virus X and 3’ truncated preS/S sequences derived from human hepatomas encode functionally active transactivators. Oncogene. (1994) 9:3335–44., PMID: 7936659

[39] Matsuoka M Jeang KT . Human T-cell leukemia virus type 1 (HTLV-1) and leukemic transformation: viral infectivity, Tax, HBZ and therapy. Oncogene. (2011) 30:1379–89. doi: 10.1038/onc.2010.537, PMID: 21119600 PMC3413891

[40] Cook LB Elemans M Rowan AG Asquith B . HTLV-1: persistence and pathogenesis. Virology. (2013) 435:131–40. doi: 10.1016/j.virol.2012.09.028, PMID: 23217623

[41] Kimura R Senba M Cutler SJ Ralph SJ Xiao G Mori N . Human T cell leukemia virus type I tax-induced IκB-ζ modulates tax-dependent and tax-independent gene expression in T cells. Neoplasia. (2013) 15:1110–24. doi: 10.1593/neo.131140, PMID: 24027435 PMC3769889

[42] Suzuki T Kitao S Matsushime H Yoshida M . HTLV-1 Tax protein interacts with cyclin-dependent kinase inhibitor p16INK4A and counteracts its inhibitory activity towards CDK4. EMBO J. (1996) 15:1607–14. doi: 10.1002/j.1460-2075.1996.tb00505.x, PMID: 8612584 PMC450070

[43] Zhao T Coutts A Xu L Yu J Ohshima K Matsuoka M . HTLV-1 bZIP factor supports proliferation of adult T cell leukemia cells through suppression of C/EBPα signaling. Retrovirology. (2013) 10:159. doi: 10.1186/1742-4690-10-159, PMID: 24359396 PMC3880043

[44] Ma G Yasunaga J Fan J Yanagawa S Matsuoka M . HTLV-1 bZIP factor dysregulates the Wnt pathways to support proliferation and migration of adult T-cell leukemia cells. Oncogene. (2013) 32:4222–30. doi: 10.1038/onc.2012.450, PMID: 23045287

[45] Schafer DF Sorrell MF . Hepatocellular carcinoma. Lancet. (1999) 353:1253–7. doi: 10.1016/S0140-6736(98)09148-X, PMID: 10217098

[46] Dionisio N Garcia-Mediavilla MV Sanchez-Campos S Majano PL Benedicto I Rosado JA . Hepatitis C virus NS5A and core proteins induce oxidative stress-mediated calcium signalling alterations in hepatocytes. J Hepatol. (2009) 50:872–82. doi: 10.1016/j.jhep.2008.12.026, PMID: 19303156

[47] Zheng Y Gao B Ye L Kong L Jing W Yang X . Hepatitis C virus non-structural protein NS4B can modulate an unfolded protein response. J Microbiol. (2005) 43:529–36., PMID: 16410770

[48] de Mochel NS Seronello S Wang SH Ito C Zheng JX Liang TJ . Hepatocyte NAD(P)H oxidases as an endogenous source of reactive oxygen species during hepatitis C virus infection. Hepatology. (2010) 52:47–59. doi: 10.1002/hep.23671, PMID: 20578128 PMC3141587

[49] Syed GH Amako Y Siddiqui A . Hepatitis C virus hijacks host lipid metabolism. Trends Endocrinol Metab. (2010) 21:33–40. doi: 10.1016/j.tem.2009.07.005, PMID: 19854061 PMC2818172

[50] Bittar C Shrivastava S Bhanja Chowdhury J Rahal P Ray RB . Hepatitis C virus NS2 protein inhibits DNA damage pathway by sequestering p53 to the cytoplasm. PloS One. (2013) 8:e62581. doi: 10.1371/journal.pone.0062581, PMID: 23638118 PMC3640050

[51] Cheng D Zhang L Yang G Zhao L Peng F Tian Y . Hepatitis C virus NS5A drives a PTEN-PI3K/Akt feedback loop to support cell survival. Liver Int. (2015) 35:1682–91. doi: 10.1111/liv.12733, PMID: 25388655

[52] Zhao LJ Wang L Ren H Cao J Li L Ke JS . Hepatitis C virus E2 protein promotes human hepatoma cell proliferation through the MAPK/ERK signaling pathway via cellular receptors. Exp Cell Res. (2005) 305:23–32. doi: 10.1016/j.yexcr.2004.12.024, PMID: 15777784

[53] Lange P Damania B . Kaposi sarcoma-associated herpesvirus (KSHV). Trends Microbiol. (2020) 28:236–7. doi: 10.1016/j.tim.2019.10.006, PMID: 31759828

[54] Sodhi A Montaner S Patel V Gómez-Román JJ Li Y Sausville EA . Akt plays a central role in sarcomagenesis induced by Kaposi’s sarcoma herpesvirus-encoded G protein-coupled receptor. Proc Natl Acad Sci U.S.A. (2004) 101:4821–6. doi: 10.1073/pnas.0400835101, PMID: 15047889 PMC387332

[55] Liu R Li X Tulpule A Zhou Y Scehnet JS Zhang S . KSHV-induced notch components render endothelial and mural cell characteristics and cell survival. Blood. (2010) 115:887–95. doi: 10.1182/blood-2009-08-236745, PMID: 19965636 PMC2815507

[56] Fujimuro M Wu FY ApRhys C Kajumbula H Young DB Hayward GS . A novel viral mechanism for dysregulation of beta-catenin in Kaposi’s sarcoma-associated herpesvirus latency. Nat Med. (2003) 9:300–6. doi: 10.1038/nm829, PMID: 12592400

[57] Sousa-Squiavinato AC Silvestre RN Elgui De Oliveira D . Biology and oncogenicity of the Kaposi sarcoma herpesvirus K1 protein. Rev Med Virol. (2015) 25:273–85. doi: 10.1002/rmv.1843, PMID: 26192396

[58] Chang HH Ganem D . A unique herpesviral transcriptional program in KSHV-infected lymphatic endothelial cells leads to mTORC1 activation and rapamycin sensitivity. Cell Host Microbe. (2013) 13:429–40. doi: 10.1016/j.chom.2013.03.009, PMID: 23601105 PMC3774835

[59] McCormick C Ganem D . The kaposin B protein of KSHV activates the p38/MK2 pathway and stabilizes cytokine mRNAs. Science. (2005) 307:739–41. doi: 10.1126/science.1105779, PMID: 15692053

[60] Chugh P Matta H Schamus S Zachariah S Kumar A Richardson JA . Constitutive NF-kappaB activation, normal Fas-induced apoptosis, and increased incidence of lymphoma in human herpes virus 8 K13 transgenic mice. Proc Natl Acad Sci U.S.A. (2005) 102:12885–90. doi: 10.1073/pnas.0408577102, PMID: 16120683 PMC1200255

[61] Fujimuro M Hayward SD . The latency-associated nuclear antigen of Kaposi’s sarcoma-associated herpesvirus manipulates the activity of glycogen synthase kinase-3beta. J Virol. (2003) 77:8019–30. doi: 10.1128/JVI.77.14.8019-8030.2003, PMID: 12829841 PMC161926

[62] Tomlinson CC Damania B . The K1 protein of Kaposi’s sarcoma-associated herpesvirus activates the Akt signaling pathway. J Virol. (2004) 78:1918–27. doi: 10.1128/JVI.78.4.1918-1927.2004, PMID: 14747556 PMC369501

[63] Becker JC Stang A DeCaprio JA Cerroni L Lebbe C Veness M . Merkel cell carcinoma. Nat Rev Dis Primers. (2017) 3:17077. doi: 10.1038/nrdp.2017.77, PMID: 29072302 PMC6054450

[64] Shuda M Kwun HJ Feng H Chang Y Moore PS . Human Merkel cell polyomavirus small T antigen is an oncoprotein targeting the 4E-BP1 translation regulator. J Clin Invest. (2011) 121:3623–34. doi: 10.1172/JCI46323, PMID: 21841310 PMC3163959

[65] Li J Wang X Diaz J Tsang SH Buck CB You J . Merkel cell polyomavirus large T antigen disrupts host genomic integrity and inhibits cellular proliferation. J Virol. (2013) 87:9173–88. doi: 10.1128/JVI.01216-13, PMID: 23760247 PMC3754048

[66] Dong L Lu D Chen R Lin Y Zhu H Zhang Z . Proteogenomic characterization identifies clinically relevant subgroup s of intrahepatic cholangiocarcinoma. Cancer Cell. (2022) 40:70–87.e15. doi: 10.1016/j.ccell.2021.12.006, PMID: 34971568

[67] Jin J Kouznetsova VL Kesari S Tsigelny IF . Synergism in actions of HBV with aflatoxin in cancer development. Toxicology. (2023) 499:153652. doi: 10.1016/j.tox.2023.153652, PMID: 37858775

[68] Lam YK Yu J Huang H Ding X Wong AM Leung HH . TP53 R249S mutation in hepatic organoids captures the predisposing can cer risk. Hepatol (Baltimore Md.). (2023) 78:727–40. doi: 10.1002/hep.32802, PMID: 36221953 PMC10086078

[69] Liu NN Yi CX Wei LQ Zhou JA Jiang T Hu CC . The intratumor mycobiome promotes lung cancer progression via myeloid-derived suppressor cells. Cancer Cell. (2023) 41:1927–1944.e9. doi: 10.1016/j.ccell.2023.08.012, PMID: 37738973

[70] Puerta-Alcalde P Monzó-Gallo P Aguilar-Guisado M Ramos JC Laporte-Amargós J MaChado M . Breakthrough invasive fungal infection among patients with haematologi c Malignancies: A national, prospective, and multicentre study. J Infect. (2023) 87:46–53. doi: 10.1016/j.jinf.2023.05.005, PMID: 37201859

[71] Lin Y Lau HC-H Liu Y Kang X Wang Y Ting NL-N . Altered Mycobiota Signatures and Enriched Pathogenic Aspergillus rambellii Are Associated With Colorectal Cancer Based on Multicohort Fecal Metagenomic Analyses. Gastroenterology. (2022) 163:908–21. doi: 10.1053/j.gastro.2022.06.038, PMID: 35724733

[72] Long X Wong CC Tong L Chu ESH Ho Szeto C Go MYY . Peptostreptococcus anaerobius promotes colorectal carcinogenesis and modulates tumour immunity. Nat Microbiol. (2019) 4:2319–30. doi: 10.1038/s41564-019-0541-3, PMID: 31501538

[73] Wang X Fang Y Liang W Cai Y Wong CC Wang J . Gut-liver translocation of pathogen Klebsiella pneumoniae promotes hepatocellular carcinoma in mice. Nat Microbiol. (2025) 10:169–84. doi: 10.1038/s41564-024-01890-9, PMID: 39747695 PMC11726454

[74] Rubinstein MR Baik JE Lagana SM Han RP Raab WJ Sahoo D . Fusobacterium nucleatum promotes colorectal cancer by inducing Wnt/β-catenin modulator Annexin A1. EMBO Rep. (2019) 20(4). doi: 10.15252/embr.201847638, PMID: 30833345 PMC6446206

[75] Sun D Yu F Ma Y Zhao R Chen X Zhu J . MicroRNA-31 activates the RAS pathway and functions as an oncogenic MicroRNA in human colorectal cancer by repressing RAS p21 GTPase activating protein 1 (RASA1). J Biol Chem. (2013) 288:9508–18. doi: 10.1074/jbc.M112.367763, PMID: 23322774 PMC3611019

[76] Sun D Wang C Long S Ma Y Guo Y Huang Z . C/EBP-β-activated microRNA-223 promotes tumour growth through targeting RASA1 in human colorectal cancer. Br J Cancer. (2015) 112:1491–500. doi: 10.1038/bjc.2015.107, PMID: 25867276 PMC4453668

[77] Zhang S Yang Y Weng W Guo B Cai G Ma Y . Fusobacterium nucleatum promotes chemoresistance to 5-fluorouracil by upregulation of BIRC3 expression in colorectal cancer. J Exp Clin Cancer Res. (2019) 38:14. doi: 10.1186/s13046-018-0985-y, PMID: 30630498 PMC6327560

[78] Yu T Guo F Yu Y Sun T Ma D Han J . Fusobacterium nucleatum promotes chemoresistance to colorectal cancer by modulating autophagy. Cell. (2017) 170:548–563.e16. doi: 10.1016/j.cell.2017.07.008, PMID: 28753429 PMC5767127

[79] Ramos A Hemann MT . Drugs, bugs, and cancer: fusobacterium nucleatum promotes chemoresistance in colorectal cancer. Cell. (2017) 170:411–3. doi: 10.1016/j.cell.2017.07.018, PMID: 28753421

[80] Lu P Xu M Xiong Z Zhou F Wang L . Fusobacterium nucleatum prevents apoptosis in colorectal cancer cells via the ANO1 pathway. Cancer Manag Res. (2019) 11:9057–66. doi: 10.2147/CMAR.S185766, PMID: 31802939 PMC6829176

[81] Gur C Maalouf N Shhadeh A Berhani O Singer BB Bachrach G . Fusobacterium nucleatum supresses anti-tumor immunity by activating CEACAM1. Oncoimmunology. (2019) 8:e1581531. doi: 10.1080/2162402X.2019.1581531, PMID: 31069151 PMC6492956

[82] Van Doorslaer K McBride AA . Molecular archeological evidence in support of the repeated loss of a papillomavirus gene. Sci Rep. (2016) 6:33028. doi: 10.1038/srep33028, PMID: 27604338 PMC5015084

[83] Buckley CE St Johnston D . Apical-basal polarity and the control of epithelial form and function. Nat Rev Mol Cell Biol. (2022) 23:559–77. doi: 10.1038/s41580-022-00465-y, PMID: 35440694

[84] Wörthmüller J Rüegg C . MAGI1, a scaffold protein with tumor suppressive and vascular functions. Cells. (2021) 10:1494. doi: 10.3390/cells10061494, PMID: 34198584 PMC8231924

[85] Zhou Y-Q Jiang J-X He S Li Y-Q Cheng X-X Liu S-Q . Epstein-Barr virus hijacks histone demethylase machinery to drive epithelial Malignancy progression through KDM5B upregulation. Signal Transduction Targeted Ther. (2025) 10:83. doi: 10.1038/s41392-025-02163-5, PMID: 40059116 PMC11891327

[86] Yang P Markowitz GJ Wang XF . The hepatitis B virus-associated tumor microenvironment in hepatocellular carcinoma. Natl Sci Rev. (2014) 1:396–412. doi: 10.1093/nsr/nwu038, PMID: 25741453 PMC4346158

[87] Ogunwobi OO Harricharran T Huaman J Galuza A Odumuwagun O Tan Y . Mechanisms of hepatocellular carcinoma progression. World J Gastroenterol. (2019) 25:2279–93. doi: 10.3748/wjg.v25.i19.2279, PMID: 31148900 PMC6529884

[88] Vona G Estepa L Béroud C Damotte D Capron F Nalpas B . Impact of cytomorphological detection of circulating tumor cells in patients with liver cancer. Hepatology. (2004) 39:792–7. doi: 10.1002/hep.20091, PMID: 14999698

[89] Nishida N Kudo M . Immunological microenvironment of hepatocellular carcinoma and its clinical implication. Oncology. (2017) 92 Suppl 1:40–9. doi: 10.1159/000451015, PMID: 27764823

[90] Fujita M Chen MM Siwak DR Sasagawa S Oosawa-Tatsuguchi A Arihiro K . Proteo-genomic characterization of virus-associated liver cancers reveals potential subtypes and therapeutic targets. Nat Commun. (2022) 13:6481. doi: 10.1038/s41467-022-34249-x, PMID: 36309506 PMC9617926

[91] Reungoat E Grigorov B Zoulim F Pécheur E-I . Molecular crosstalk between the hepatitis C virus and the extracellular matrix in liver fibrogenesis and early carcinogenesis. Cancers. (2021) 13:2270. doi: 10.3390/cancers13092270, PMID: 34065048 PMC8125929

[92] Cuesta Á.M Palao N Bragado P Gutierrez-Uzquiza A Herrera B Sánchez A . New and old key players in liver cancer. Int J Mol Sci. (2023) 24:17152. doi: 10.3390/ijms242417152, PMID: 38138981 PMC10742790

[93] Hofmann M Tauber C Hensel N Thimme R . CD8+ T cell responses during HCV infection and HCC. J Clin Med. (2021) 10:991. doi: 10.3390/jcm10050991, PMID: 33801203 PMC7957882

[94] Thimme R . T cell immunity to hepatitis C virus: Lessons for a prophylactic vaccine. J Hepatol. (2021) 74:220–9. doi: 10.1016/j.jhep.2020.09.022, PMID: 33002569

[95] McLane LM Abdel-Hakeem MS Wherry EJ . CD8 T cell exhaustion during chronic viral infection and cancer. Annu Rev Immunol. (2019) 37:457–95. doi: 10.1146/annurev-immunol-041015-055318, PMID: 30676822

[96] Li S Bai L Dong J Sun R Lan K . Kaposi’s sarcoma-associated herpesvirus: epidemiology and molecular biology. In: Cai Q Yuan Z Lan K , editors. nfectious Agents Associated Cancers: Epidemiology and Molecular Biology. Springer Singapore, Singapore (2017). p. 91–127. 10.1007/978-981-10-5765-6_7 29052134

[97] Cesarman E Chadburn A Rubinstein PG . KSHV/HHV8-mediated hematologic diseases. Blood. (2022) 139:1013–25. doi: 10.1182/blood.2020005470, PMID: 34479367 PMC8854683

[98] Goncalves PH Ziegelbauer J Uldrick TS Yarchoan R . Kaposi sarcoma herpesvirus-associated cancers and related diseases. Curr Opin HIV AIDS. (2017) 12:47–56. doi: 10.1097/COH.0000000000000330, PMID: 27662501 PMC6311702

[99] Czech-Sioli M Günther T Therre M Spohn M Indenbirken D Theiss J . High-resolution analysis of Merkel Cell Polyomavirus in Merkel Cell Carcinoma reveals distinct integration patterns and suggests NHEJ and MMBIR as underlying mechanisms. PloS Pathog. (2020) 16:e1008562. doi: 10.1371/journal.ppat.1008562, PMID: 32833988 PMC7470373

[100] Feng H Shuda M Chang Y Moore PS . Clonal integration of a polyomavirus in human Merkel cell carcinoma. Science. (2008) 319:1096–100. doi: 10.1126/science.1152586, PMID: 18202256 PMC2740911

[101] Borchert S Czech-Sioli M Neumann F Schmidt C Wimmer P Dobner T . High-affinity Rb binding, p53 inhibition, subcellular localization, and transformation by wild-type or tumor-derived shortened Merkel cell polyomavirus large T antigens. J Virol. (2014) 88:3144–60. doi: 10.1128/JVI.02916-13, PMID: 24371076 PMC3957953

[102] Houben R Adam C Baeurle A Hesbacher S Grimm J Angermeyer S . An intact retinoblastoma protein-binding site in Merkel cell polyomavirus large T antigen is required for promoting growth of Merkel cell carcinoma cells. Int J Cancer. (2012) 130:847–56. doi: 10.1002/ijc.26076, PMID: 21413015

[103] Sastre-Garau X Peter M Avril MF Laude H Couturier J Rozenberg F . Merkel cell carcinoma of the skin: pathological and molecular evidence for a causative role of MCV in oncogenesis. J Pathol. (2009) 218:48–56. doi: 10.1002/path.2532, PMID: 19291712

[104] Shuda M Feng H Kwun HJ Rosen ST Gjoerup O Moore PS . T antigen mutations are a human tumor-specific signature for Merkel cell polyomavirus. Proc Natl Acad Sci U.S.A. (2008) 105:16272–7. doi: 10.1073/pnas.0806526105, PMID: 18812503 PMC2551627

[105] Houben R Shuda M Weinkam R Schrama D Feng H Chang Y . Merkel cell polyomavirus-infected Merkel cell carcinoma cells require expression of viral T antigens. J Virol. (2010) 84:7064–72. doi: 10.1128/JVI.02400-09, PMID: 20444890 PMC2898224

[106] Verhaegen ME Mangelberger D Harms PW Vozheiko TD Weick JW Wilbert DM . Merkel cell polyomavirus small T antigen is oncogenic in transgenic mice. J Invest Dermatol. (2015) 135:1415–24. doi: 10.1038/jid.2014.446, PMID: 25313532 PMC4397111

[107] Hernández-Ramírez RU Shiels MS Dubrow R Engels EA . Cancer risk in HIV-infected people in the USA from 1996 to 2012: a population-based, registry-linkage study. Lancet HIV. (2017) 4:e495–504. doi: 10.1016/S2352-3018(17)30125-X, PMID: 28803888 PMC5669995

[108] Pantanowitz L Carbone A Dolcetti R . Microenvironment and HIV-related lymphomagenesis. Semin Cancer Biol. (2015) 34:52–7. doi: 10.1016/j.semcancer.2015.06.002, PMID: 26118690

[109] Kundu RK Sangiorgi F Wu L-Y Pattengale PK Hinton DR Gill PS . Expression of the human immunodeficiency virus-tat gene in lymphoid tissues of transgenic mice is associated with B-cell lymphoma. Blood. (1999) 94:275–82. doi: 10.1182/blood.V94.1.275.413a30_275_282, PMID: 10381523

[110] Lazzi S Bellan C De Falco G Cinti C Ferrari F Nyongo A . Expression of RB2/p130 tumor-suppressor gene in AIDS-related non-Hodgkin’s lymphomas: Implications for disease pathogenesis. Hum Pathol. (2002) 33:723–31. doi: 10.1053/hupa.2002.125372, PMID: 12196924

[111] Scala G Ruocco MR Ambrosino C Mallardo M Giordano V Baldassarre F . The expression of the interleukin 6 gene is induced by the human immunodeficiency virus 1 TAT protein. The Journal of experimental medicine (1994) 179:961–71. doi: 10.1084/jem.179.3.961, PMID: 8113688 PMC2191426

[112] Blazevic V Heino M Lagerstedt A Ranki A Krohn KJ . Interleukin-10 gene expression induced by HIV-1 Tat and Rev in the cells of HIV-1 infected individuals. Journal of acquired immune deficiency syndromes and human retrovirology : official publication of the International Retrovirology Association (1996) 13:208–14. doi: 10.1097/00042560-199611010-00002, PMID: 8898665

[113] Srivastava DK Tendler CL Milani D English MA Licht JD Wilson SHJA . The HIV-1 transactivator protein Tat is a potent inducer of the human DNA repair enzyme β-polymerase. AIDS (London, England) (2001) 15:433–40. doi: 10.1097/00002030-200103090-00001, PMID: 11242139

[114] Rusnati M Presta MJA . HIV-1 Tat protein and endothelium: from protein/cell interaction to AIDS-associated pathologies. Angiogenesis (2002) 5:141–51. doi: 10.1023/A:1023892223074, PMID: 12831055

[115] Shiramizu B Herndier BG McGrath MS . Identification of a common clonal human immunodeficiency virus integration site in human immunodeficiency virus-associated lymphomas. Cancer research (1994) 54:2069–72., PMID: 8174106

[116] Qin Y Zhang L Xu Z Zhang J Jiang Y-Y Cao Y . Innate immune cell response upon Candida albicans infection. Virulence. (2016) 7:512–26. doi: 10.1080/21505594.2016.1138201, PMID: 27078171 PMC5026795

[117] Cullin N Azevedo Antunes C Straussman R Stein-Thoeringer CK Elinav E . Microbiome and cancer. Cancer Cell. 39:1317–41. doi: 10.1016/j.ccell.2021.08.006, PMID: 34506740

[118] Vallianou N Kounatidis D Christodoulatos GS Panagopoulos F Karampela I Dalamaga M . Mycobiome and cancer: what is the evidence? Cancers. (2021) 13:3149. doi: 10.3390/cancers13133149, PMID: 34202433 PMC8269322

[119] Aykut B Pushalkar S Chen R Li Q Abengozar R Kim JI . The fungal mycobiome promotes pancreatic oncogenesis via activation of MBL. Nature. (2019) 574:264–7. doi: 10.1038/s41586-019-1608-2, PMID: 31578522 PMC6858566

[120] Duong MT Qin Y You SH Min JJ . Bacteria-cancer interactions: bacteria-based cancer therapy. Exp Mol Med. (2019) 51:1–15. doi: 10.1038/s12276-019-0297-0, PMID: 31827064 PMC6906302

[121] Saftien A Puschhof J Elinav E . Fungi and cancer. Gut. (2023) 72:1410–25. doi: 10.1136/gutjnl-2022-327952, PMID: 37147013

